# Stability and Change of Individual Differences in Ideal Partner Preferences Over 13 Years

**DOI:** 10.1177/01461672231164757

**Published:** 2023-04-08

**Authors:** Julie C. Driebe, Julia Stern, Lars Penke, Tanja M. Gerlach

**Affiliations:** 1 University of Goettingen, Germany; 2 University of Bremen, Germany; 3 Leibniz ScienceCampus Primate Cognition, Goettingen, Germany; 4 Queen’s University Belfast, UK

**Keywords:** romantic relationships, ideal partner preferences, development, perceived change, insight

## Abstract

Ideal partner preferences for traits in a partner are said to be stable cognitive constructs. However, longitudinal studies investigating the same participants’ ideals repeatedly have so far been limited to relatively short retest intervals of a maximum of 3 years. Here, we investigate the stability and change of ideals across 13 years and participants’ insight into how ideals have changed. A total of 204 participants (*M* = 46.2 years, *SD* = 7.4, 104 women) reported their ideals at two time points. We found a mean rank-order stability of *r* = .42 and an overall profile stability of *r* = .73 (distinctive *r* = .53). Some ideals changed over time, for example, increased for status-resources in relation to age and parenthood. We found some but varying insight into how ideals had changed (mean *r* = .20). Results support the idea of ideals being stable cognitive constructs but suggest some variability related to the demands of different life stages.

Popular culture suggests that when considering the traits they would like to have in a potential romantic partner, people have a certain “type” they go after: That is, people desire and prefer specific attributes in a romantic partner, and these preferences are also thought to persist over time. However, after experiencing certain life events such as break-ups with a partner previously seen as ideal or growing feelings of immediacy to have children, people may feel that their ideas about the right person have changed. They may have learned what they cannot put up with in a partner during a romantic relationship or have come to think differently about what kind of partner they would need for the next step in their life to happen. So far, very little is known about how stable or malleable peoples’ ideas about the right person are over extended periods of time. In addition, it is unclear whether people are able to track if their preferences for a romantic partner have changed. Hence, in the current study, we investigate the stability and change of ideal partner preferences across a timespan of 13 years and explore whether individuals possess insight into how their preferences have changed.

## Ideal Partner Preferences

With ideal partner preferences, we refer to the aspirations or standards an individual has about an ideal partner (Simpson et al., 2001). These standards are used to evaluate (potential) partners and should thereby guide relationship decisions (Fletcher et al., 1999; Simpson et al., 2001). The Ideal Standards Model (ISM) provides a framework to describe the qualities of an ideal partner using three correlated factors: Warmth-trustworthiness, vitality-attractiveness, and status-resources (Fletcher et al., 1999). These three factors are well-replicated (Campbell & Fletcher, 2015). However, as suggested by other studies (e.g., Shackelford et al., 2005b), qualities describing an ideal partner may not be limited to these three factors. Other factors that have been reported include, for example, family orientation (Lam et al., 2016); intellect, dominance (Csajbók & Berkics, 2017; Schwarz & Hassebrauck, 2012); or humor and sociability (Schwarz & Hassebrauck, 2012). The ISM proposes that interindividual differences may exist since each preference dimension corresponds to a different route potentially increasing an individual’s reproductive success (Fletcher et al., 1999, 2020). A preference for warmth-trustworthiness should facilitate the formation of a secure and reliable relationship. A high preference for vitality-attractiveness should be helpful in acquiring a younger and healthy partner and, thus, be related to increased partner fertility and offspring genetic quality. Placing higher importance on status-resources should orient individuals toward partners with a higher position in social hierarchies, thereby granting greater access to resources and potentially increasing chances of offspring survival (Buss, 1989; cf. Simpson et al., 2001). Research has found that women compared to men place higher importance on status-resources and less importance on vitality-attractiveness (for an overview see Campbell & Fletcher, 2015). Furthermore, interindividual differences in preferences may be maintained since individuals are attracted toward similar others (Byrne & Blaylock, 1963), which results in couples who resemble each other in their intelligence or personality (Luo, 2017; Watson et al., 2004).

Although Fletcher and colleagues (1999) defined ideal partner preferences as stable cognitive constructs which differ between individuals, evidence on the stability of these preferences is scarce. The largest retest stability so far was found after a period of 3 weeks, *r* = .85^1^ (Fletcher et al., 1999), and 3 months, *r* = .82^1^ (Fletcher et al., 2000). Retest correlations seem to reduce substantially as more time goes by, for example, to *r* = .65 after 5 months (Gerlach et al., 2019), *r* = .55^1^ after 27 months (Bredow & Hames, 2019), and *r* = .51^1^ (Shackelford et al., 2005a) or *r* = .35 after 3 years (Bleske-Rechek & Ryan, 2015).^2^ Yet, it remains unclear whether ideal partner preferences are stable over a longer time span. If ideal partner preferences are stable, the strongest declines in stability coefficients may be found in the first years after assessment and stabilize thereafter, similar to stability in personality traits (Anusic & Schimmack, 2016; Costa et al., 2019). To our knowledge, so far, no study investigated how stable ideals are for a timespan exceeding 3 years. If these standards used to evaluate partners are indeed stable constructs, then they should show substantial stability even over an extended period of time.

However, there is also reason to believe that ideals change over a longer time span, that is, that stability should decrease after more and more years. For example, life events such as marriage, but also romantic relationships in general, are discussed to be associated with changes in personality traits (Bleidorn et al., 2018). For changes in ideal partner preferences, similar events may be associated with intraindividual changes. Developmental theories on human motivation posit that individuals face different challenges during life that go along with a shift in life priorities (Heckhausen et al., 2010, 2019). Life History Theory (LHT) may offer a rationale for changes we may observe in ideal partner preferences. LHT proposes that every individual has a limited budget of effort and resources (Alexander, 1987; Del Giudice et al., 2016). Across the lifespan, individuals should therefore face trade-offs in what kind of activities they allocate their energy to, with one trade-off existing between parental and mating effort (Del Giudice et al., 2016). Accordingly, individuals could experience shifts in their ideals related to the demands of different life stages. For example, a preference for attractiveness could be especially important during life stages where much effort is allocated to mating, and finding a young and healthy partner could increase the offspring’s quality (Buss & Schmitt, 1993; Fletcher et al., 1999). A preference for resources could be especially important during stages in life at which more effort is allocated to parenting when more resources are needed to provide care for offspring (Campbell & Fletcher, 2015). Because secure relationships characterized by high levels of cooperation and support should always be beneficial, the preference for warmth-trustworthiness might be less susceptible to lifespan-related changes. In sum, preference change for some dimensions might reflect shifting life demands and priorities as suggested by LHT. Nevertheless, as ideas borrowed from LHT may only speak to some preference dimensions but not to others, and there generally is a lack of previous research investigating changes in ideal partner preferences over a more extended period of time, the current study sets out to close this gap in the preference literature with a rather exploratory investigation.

As aforementioned, the ISM proposes that ideal partner preferences are used to evaluate potential partners. However, findings on whether participants’ ideal partner preferences are associated with their actual mate choices are inconsistent. While some studies suggest preferences play a role in peoples’ mating decisions (e.g., Campbell et al., 2016; Conroy-Beam & Buss, 2016; Gerlach et al., 2019; Park & MacDonald, 2019), other studies have concluded that preferences play no or only a negligible role for actual mate choices (e.g., Eastwick & Finkel, 2008; Eastwick et al., 2019; Joel et al., 2017; Todd et al., 2007). Here, we refrain from diving deeper into this debate as it is not the focus of the current study’s investigation. However, the answer of whether ideal partner preferences are stable or changing over an extended period of time has important implications for future research investigating the role of preferences on mating decisions. For example, if partner preferences were constantly changing, an altered version of preferences might be associated with relationship decisions. This in turn could explain the mixed findings in previous research.

## Insight Into Changes in Ideal Partner Preferences

People may have opinions on whether they prefer the same type of partner as they already did a decade ago, or whether their preferences from back then have changed. However, it is unclear whether these opinions are accurate. Two previous studies addressed perceptions of change in ideal partner preferences. Sprecher et al. (2018) asked 738 single participants aged 18 to 40 how they believed to have changed across 2 to 3 years in their preferences for a physically attractive partner, a partner’s status and resources, his or her social network, and intrinsic characteristics. In the Sprecher et al. (2018) sample, participants believed to place higher importance on all dimensions compared with earlier in life, whereas older individuals assumed to have changed to a smaller degree than younger individuals. One exception was physical attractiveness: Women, but not men, believed that they would value physical attractiveness less as compared with 2 to 3 years earlier in their life. Bleske-Rechek and colleagues (2009) asked 104 students from 18 to 25 how they believe ideals would change during college. Participants assumed an increased interest in long-term relationships, at the cost of a decreased interest in short-term relationships, and expected a partner’s personality to become more, but appears to become less important. In additional samples, the authors then investigated whether these beliefs mapped onto differences in ideals across different age groups. Results showed that, corresponding to expected changes, a partner’s personality became more and appearance became less important with increasing age in a heterogeneous sample with a wide age range but not in a student sample. Hence, previous studies either investigated (a) participants’ actual preference changes or (b) how participants believed to have changed. However, none of these studies directly investigated whether these perceived changes correspond to actual changes in ideals—in fact a person’s perception of changes may be biased (e.g., due to recall biases). Given that such changes are an intraindividual process, a more direct approach to investigate insight into preference change would be a longitudinal design.

## The Current Project

In the current study, we followed up on individuals from a former study that assessed participants’ ideal partner preferences (among other measures) in the year 2006. Approximately 13 years later, we assessed these ideals for a second time and investigated whether participants perceived that their preferences had changed over time. Hence, the current study uses a longitudinal design and thus stands in contrast to cross-sectional designs or retrospective reports that may be more prone to biases.

As there are different approaches to investigate the stability of ideals that come with different benefits, we investigate multiple stability indices. Rank-order stability estimates the degree to which the relative position of each individual in a population remains the same across time (Roberts & DelVecchio, 2000), one trait at a time. In contrast, profile correlations speak to the stability of a person’s whole trait profile. Finally, when investigating mean-level stability, the average change of a trait in a population is examined across time, which indicates the direction of change (Asendorpf & Neyer, 2012). Thus, while analyses of mean-level change address average changes in a population, rank-order stability concerns the stability of individual differences in a psychological construct.

We expect that ideals are stable over 13 years and predict that initial ideal partner preferences correlate positively with current partner preferences (Hypothesis 1). However, the exact magnitude of these retest correlation coefficients remains an empirical question to which, to the best of our knowledge, the current study will provide a first estimate. In terms of mean-level changes, we predict that the average preference for status-resources (Hypothesis 2) and family orientation (Hypothesis 3) increases from T1 to T2, especially when participants were younger at T1 (Hypothesis 2.1 and Hypothesis 3.1). Moreover, we predict that the desire for status-resources changes with the immediacy of a desire for or the actual existence of children (Hypothesis 4). Hence, the correlation of initial with current ideal partner preferences for status-resources should be smaller in a subsample of participants who now have children or are currently planning to have children compared with participants without children. Our rationale for these hypotheses is that at the normative age of family planning (compared with the stage when participants were initially recruited), more effort is allocated to parenting and securing resources to provide for offspring (Campbell & Fletcher, 2015). Finally, we predict that people’s perceptions of change in their ideals correspond to their actual changes in preferences for status-resources (Hypothesis 5.1) and vitality-attractiveness (Hypothesis 5.2). People may have more insight into changes in these two dimensions as Bleske-Rechek et al. (2009) found that participants believed external characteristics (e.g., appearance) to become less important with age.^3^

## Method

Our preregistration can be found on the OSF (https://osf.io/x7rma), next to our codebook, data, and code (https://osf.io/z6yaj). Because we deviated from our preregistered analyses, only our study design and hypotheses should be regarded as preregistered. In the following, we report all exclusions and all measures that were part of the study as well as the reasons for deviations, where applicable.

### Participants and Recruitment

We rerecruited participants of the Berlin Speed Dating Study (BSDS) that was conducted in 2006 (Asendorpf et al., 2011). This study consisted of 382 participants (age *M* = 32.8 years, *SD* = 7.4, range = 18–54, female = 192, male = 190) which we tried to contact after around 13 years. At the initial assessment (T1), participants gave us detailed contact information. From February to November 2019, we reached out to those former participants for a reassessment (T2). As an incentive, participants received individual feedback on their personality, a comparison of their initial and current ideals, and 40€, with the chance to receive a bonus of 10€.^4^

We were able to recruit 226 participants (41% dropout) of the BSDS. We excluded four participants because they reported a non-heterosexual orientation at T2 (since rated ideal partner preferences at T1 were made for the other sex) and 18 participants who dropped out during the T2 assessment. Our final sample size comprised *N* = 204 participants (age *M* = 46.2 years, *SD* = 7.4, range = 31–66; female = 104, male = 100). At T1, none of the BSDS participants were in a romantic relationship (0%). At T2, upon reassessment for the current study, 64% of participants were in a romantic relationship, 35% were single, and less than 1% reported an undefined relationship status. In all, 84% of participants reported having a university degree. An attrition analysis revealed that participants who completed participation at T2 were less conscientious (rerecruited participants *M* = 3.77 [0.62], dropout *M* = 3.89 [0.60], *p* = .05 Hedges, *g* = −0.20) and more neurotic (rerecruited participants *M* = 2.69 [0.72], dropout *M* = 2.52 [0.70], *p* = .03, Hedges *g* = 0.23) compared with participants who participated only at T1. We found no other systematic group differences in demographics and other personality traits (see Supplemental Material S1).

Because we could not foresee how many people we would be able to rerecruit for T2, we invited participants from another earlier study, the Sociosexuality Study, taking place between 2004 and 2005 (Penke & Asendorpf, 2008) to increase our chance of a sufficient sample size.^5^ However, deviating from our preregistration, we decided to run our main analyses based on participants of the BSDS only and include analyses of this second sample in our Supplemental Material because this sample came with several major limitations^6^ and was difficult to compare with the BSDS reassessment yet turned out to be too small to analyze on its own (see Supplemental Material S1B, S3).

### Procedure

The reassessment was implemented on the survey framework formr.org (Arslan et al., 2020). We invited participants to our online study with the aim to investigate their relationship history longitudinally. After participants agreed to participate, they were asked to fill out a short demographic questionnaire including questions on their age, sex, relationship status, and family situation (Table 1). In the second step, participants were asked to rate various items regarding their importance in an ideal partner. In the third step, participants were introduced to an event-history calendar in which they were asked to fill in all places they had lived in and occupations they had held as well as life events that they considered to be important to them. In this calendar, places of living and occupations held served as retrieval cues to accurately remember all relationships participants had entered (Belli et al., 2001; Tully & Meyvis, 2017). Hence, participants were asked to fill in all relationships that lasted longer than 6 months as well as their current relationship, independent of its length. In a fourth step, a detailed assessment of participants’ relationship histories followed; however, this assessment is beyond the scope of the current investigation and is reported in more detail in a different manuscript (Driebe et al., 2023). In a fifth step, participants finished their participation by answering different measures on their personalities including questions on how they perceive their ideals had changed since their initial participation. Although not part of the current study, participants were finally asked to invite peers and their current partners to participate in a second part of the reassessment.

**Table 1.** Item Content and Response Formats of Demographic Questionnaire Used in This Study.

Item	Content [Response format]
Sex	Your biological sex: [female, male]
Age	Your current age: [number between 18 to 100]
Relationship Status	What is your relationship status? [Single, uncommitted relationship, committed relationship, engaged, married, other]
Children	Do you have children? [Yes, No]
No. of children^a^	How many biological/adopted children do you have? [number between 1 and 40]
Wish for children^b^	Do you ever want to have children? [Yes, No, Do not know yet]
Pregnant trying	Do you currently try to become a father/mother (again)? [1: try to avoid it - 5: trying]

a The item was only presented to participants who indicated to have children. ^b^The item was only presented to participants who indicated not to have children.

### Measures

#### Ideal Partner Preferences

At T1 and T2, participants rated the same 58 characteristics^7^ on their importance in an ideal partner on a scale ranging from 1 (*very unimportant)* to 5 (*very important)*. However, at T2, we specified to rate a partner for a committed, long-term relationship and if participants were currently involved in a romantic relationship, it was noted to make each rating independent of one’s current partner. Since at T1 none of the participants were involved in a romantic relationship, the additional instruction on making one’s rating independent of one’s current partner was not needed.

In a principal component analysis with oblimin rotation from a pretest conducted in another sample (see Supplemental Material S1C) before our T2 survey was set up, we extracted eight factors, which we labeled warmth-trustworthiness, vitality-attractiveness, status-resources, family orientation, intelligence, creativity, humor, and adventurousness-confidence. We preregistered to use these eight preference dimensions for our analyses on our actual sample. Hence, Table 2 entails descriptive data of all items and dimensions assessing participants’ ideal partner preferences at T1 and T2.

**Table 2.** Cronbach’s α, Means and Standard Deviations of All Items Assessing Ideal Partner Preferences at T1 and T2.

Item of each dimension	*a_T1_*	*M_T1_ (SD_T1_)*	*a_T2_*	*M_T2_ (SD_T2_)*
Warmth-trustworthiness	0.82		0.90	
• Understanding		4.15 (0.67)		4.28 (0.78)
• Sensitive		4.22 (0.63)		4.27 (0.70)
• Trustworthy		4.45 (0.61)		4.52 (0.66)
• Good listener		4.07 (0.72)		4.19 (0.82)
• Honest		4.67 (0.55)		4.56 (0.67)
• Considerate		3.97 (0.68)		4.20 (0.77)
• Supportive		3.79 (0.75)		4.14 (0.83)
• Faithful		4.39 (0.76)		4.22 (0.94)
• Kind		3.74 (0.96)		3.89 (0.99)
Vitality-attractiveness	0.80		0.85	
• Erotic		3.82 (0.72)		3.69 (0.87)
• Sexy		3.57 (0.91)		3.50 (0.89)
• Arousing		3.90 (0.77)		3.54 (0.88)
• Attractive		3.91 (0.74)		3.77 (0.81)
• Nice body		3.51 (0.85)		3.51 (0.90)
• Appealing		4.05 (0.68)		3.96 (0.74)
• Passionate		3.92 (0.76)		3.63 (0.88)
• Sporty and athletic		3.31 (0.93)		3.37 (0.91)
• Fit		3.49 (0.82)		3.62 (0.83)
• Feminine		2.88 (1.36)		2.87 (1.25)
Status-resources	0.84		0.85	
• Prosperous		2.12 (0.94)		2.45 (0.95)
• Good job		2.92 (0.92)		3.10 (0.92)
• Financially secure		2.83 (1.05)		3.06 (1.07)
• Successful		2.81 (0.90)		2.97 (0.92)
• Influential		2.19 (0.90)		2.28 (0.91)
• Of good standing		2.45 (1.02)		2.55 (0.93)
• Good family background		2.01 (1.01)		2.14 (1.05)
• Nice house or apartment		2.67 (1.06)		2.99 (1.01)
• Dresses well		3.59 (0.91)		3.44 (0.91)
• Healthy		3.82 (0.81)		3.81 (0.88)
Family orientation	0.86		0.90	
• Wanting to have children		3.36 (1.31)		3.12 (1.52)
• Being a good mother/father		3.42 (1.13)		3.67 (1.30)
• Likes children		3.71 (1.07)		3.72 (1.21)
• Family-oriented		3.39 (0.99)		3.61 (1.20)
Intelligence	0.72		0.78	
• Intelligent		4.34 (0.68)		4.29 (0.67)
• Educated		4.19 (0.69)		4.15 (0.79)
• Sharp		3.56 (0.87)		3.53 (0.92)
• Clever		3.42 (0.96)		3.45 (0.93)
• Eloquent		3.62 (0.87)		3.69 (0.90)
• Interesting		4.19 (0.69)		4.03 (0.76)
Creativity	0.63		0.74	
• Creative		3.55 (0.87)		3.52 (0.86)
• Broad-minded		3.21 (0.93)		3.13 (0.93)
• Inventive		3.94 (0.67)		3.65 (0.86)
• Original		3.38 (0.84)		3.42 (0.90)
Humor	0.72		0.77	
• Fun		3.74 (0.86)		3.76 (0.94)
• Good fun		3.83 (0.80)		3.79 (0.92)
• Good sense of humor		4.43 (0.68)		4.16 (0.81)
• Shrewd		3.11 (1.02)		3.45 (0.92)
• Smart		1.77 (0.88)		2.05 (0.96)
• Outgoing		3.57 (0.70)		3.48 (0.81)
Adventurousness-confidence	0.73		0.77	
• Adventurous		3.27 (0.93)		3.35 (0.96)
• Venturesome		2.74 (0.90)		2.70 (0.97)
• Masculine		2.52 (1.28)		2.50 (1.25)
• Assertive		3.59 (0.76)		3.24 (0.86)
• Self-aware		3.83 (0.70)		3.69 (0.80)
• Ambitious		3.55 (0.75)		3.60 (0.80)
• Energetic		2.83 (0.90)		2.83 (0.94)
• Confident		3.87 (0.70)		3.74 (0.75)
• Dominance		2.22 (0.93)		2.27 (0.95)

#### Perceived Change of Ideal Partner Preferences

To assess how participants perceived that their ideals had changed over time, we presented participants with the date of their initial participation (MM/YYYY) as well as their event-history calendar. After presenting these retrieval cues, we then instructed participants to think back to the time when they first participated in the study to remember exactly what was important to them in an ideal partner at that time. We then asked participants to compare former thoughts and attitudes toward an ideal partner with their current perspective. We presented eight different preference dimensions (corresponding to the factors extracted from a pre-test, see S1C) which were illustrated with four attributes each (Supplemental Table S4). For each of the eight 1-item measures for the respective preference dimension, participants indicated whether they perceived that their preferences had changed on a 5-point Likert-type scale ranging from −2 (*a lot less important than earlier*) to +2 (*a lot more important than earlier*).

#### Number of Relationships

Inspired by a reviewer’s comment, as a further exploration, we investigated stability and change in regard to how many relationships participants had entered in the time between T1 and T2, as studies have shown that individuals adjust their ideal partner preferences according to their partner (e.g., Fletcher et al., 2000; Gerlach et al., 2019). We grouped participants into three groups: participants who did not enter any relationship lasting longer than 6 months over the investigated timespan (*n* = 25), participants who entered one relationship lasting longer than 6 months (*n* = 94), and participants who entered more than one relationship lasting longer than 6 months (*n* = 85). See S2I for more details on these additional analyses.

## Results

We decided to only interpret results based on the reassessment of BSDS participants and not on both samples that had been rerecruited (i.e., the BSDS and the second sample reported in our Supplemental Material) because the second sample came with several limitations (e.g., insufficient assessment of ideal partner preferences at T1). In our Supplemental Material (S4), we provide a table with more detailed explanations for three deviations of the current analyses from our preregistered analysis plan. Because we were not able to exactly follow our preregistered analyses, only our design and hypotheses should be regarded as preregistered. Our analyses were run using R (Version 4.0.2; R Core Team, 2020), using the packages psych (Version 2.0.12; Revelle, 2020), effsize (Version 0.8.1; Torchiano, 2020), multicon (Version 1.6; Sherman, 2015), and lmerTest (Version 3.1-2; Kuznetsova et al., 2017), sjPlot (Version 2.8.7; Lüdecke, 2021).

First, we investigated the stability of ideal partner preferences. We had several approaches to investigate stability. We decided to calculate rank-order stabilities of T1 and T2 partner preferences, that is, the stability of one trait at a time, as well as profile correlations, that is, the stability of a person’s trait profile. Second, we investigated changes in ideal partner preferences by calculating mean-level changes of T1 to T2 partner preferences, which estimates the average change in a trait across a whole population. Third, we investigated the association of age with mean-level changes and the stability of ideal partner preferences when having children. Finally, we investigated whether participants have an insight into how their preferences have changed, that is, whether actual changes in partner preferences correlate with participants’ self-perceived changes in preferences. As a further exploration, we investigated potential sex differences and the number of relationships entered. For our preregistered analyses, we interpreted results with *p* < .05 as significant findings. For all exploratory analyses which were not preregistered, we used a more conservative interpretation with *p* < .01 interpreted as significant findings to correct for alpha error inflation due to multiple testing.

### Rank-Order Stability

To estimate the stability of ideal partner preferences across 13 years which we had assumed to be substantial in our first hypothesis (H1), we correlated participants’ initial (T1) with their current preferences (T2) using a Pearson product-moment correlation, separately for the eight preference dimensions (Table 3). Coefficients ranged from *r* = .31 (for warmth-trustworthiness) to *r* = .47 (for status-resources and adventurousness-confidence), with a mean correlation of *r* = .42. These results suggest substantial positive correlations between T1 and T2 preferences, supporting H1. We found no significant difference in sex difference between men’s (*r* = .44) and women’s (*r* =.36) mean correlation (*p* = .502, Supplemental Table S7). We also found no significant difference (*p* = .089) in participants overall rank-order stability depending on how many romantic relationships they had entered (Supplemental Table S20).

**Table 3.** Means, Standard Deviations, Effect Sizes, and Correlations of T1 and T2 Importance Ratings.

Preference dimension	*M_T1_ (SD_T1_)*	*M_T2_ (SD_T2_)*	*p*	*d*	*r* [95% CI]
Warmth-trustworthiness	4.16 (0.45)	4.25 (0.59)	.035	0.17 [0.01, 0.34]	.31 [.19, .43]
Vitality-attractiveness	3.64 (0.53)	3.55 (0.59)	.030	−0.16 [−0.31, −0.02]	.44 [.32, .55]
Status-resources	2.74 (0.61)	2.88 (0.62)	.002	0.22 [0.08, 0.37]	.47 [.36, .57]
Family orientation	3.47 (0.95)	3.53 (1.16)	.452	0.05 [−0.09, 0.20]	.46 [.34, .56]
Intelligence	3.89 (0.52)	3.86 (0.58)	.521	−0.05 [−0.20, 0.10]	.37 [.25, .49]
Creativity	3.52 (0.57)	3.43 (0.67)	.071	−0.14 [−0.29, 0.01]	.40 [.27, .51]
Humor	3.41 (0.53)	3.45 (0.61)	.366	0.07 [−0.08, 0.22]	.40 [.27, .51]
Adventurousness-confidence	3.16 (0.50)	3.10 (0.55)	.143	−0.11 [−0.25, 0.04]	.47 [.35, .57]

*Note. p* = *p* values of two-sided *t* tests in which we compared participant’s mean preferences at T1 and T2. CI = confidence interval.

### Profile Correlations

For further exploration of our first hypothesis on the stability of ideal partner preferences (H1), we calculated the profile correlation between T1 and T2 ideals across all dimensions. Profile correlations quantify the stability of a person’s trait profile over time. We would expect to attain high coefficients, if, for example, at T1 a person rated warmth-trustworthiness of an ideal partner as highly important and status-resources or intelligence as less important, and the traits’ rank order remained similar when rated at T2. Likewise, we would attain lower coefficients, if, at T2, this person rated, for example, intelligence as highly important in an ideal partner, and status-resources and warmth-trustworthiness as less important. We found an overall correlation of *r* = .73 (*p* < .001). This overall correlation reveals that the pattern of which traits were considered more or less important showed high temporal consistency. Because a substantial part of this association could be due to a normative component of preferences, we also investigated the distinctive profile correlation. Here, an average profile of T1 and T2 preferences is calculated, regressed on each individual profile, and the residuals of these regressions are then correlated. The distinctive stability was somewhat smaller compared with the overall profile correlation yet still considerable in magnitude (*r* = .53, *p* < .001). In addition, we also calculated the profile correlation between T1 and T2 preferences across all items (overall correlation: *r* = .62, *p* <.001; distinctive stability: *r* = .40, *p* < .001). Not surprisingly, these coefficients are somewhat smaller compared with the coefficients on a dimension level because aggregation across individual items on a dimension level reduces measurement error. Nevertheless, even on an item level, we attained very substantial correlation coefficients. Results were comparable for men and women (see Supplemental Material S2B) and also did not differ depending on how many relationships participants had entered (Supplemental Material S2I). These results corroborate our finding that ideals possess considerable stability from T1 to T2.

### Mean-Level Changes in Ideal Partner Preferences

Next, we explored mean-level changes for each dimension using paired sample tests (two-sided) to see the direction of changes as we predicted increases in the dimension status-resources (H2) and family-orientation (H3) from T1 to T2. We found significant increases in participants’ preferences for the dimensions warmth-trustworthiness and status-resources and decreases for vitality-attractiveness over time. We found no other significant mean-level changes (Table 3). These results support H2 in that participants’ preference for status-resources increased over time, but not H3, as there was no increase for family orientation.

Exploration of sex differences (Supplemental Material S2C) revealed that overall, women reported significantly higher ideals (*b* = 0.12, 95% confidence interval [CI]: [0.02, 0.21], *p* = .017). On the specific dimensions (Supplemental Material S2C), women reported a higher preference than men for status-resources (*b* = 0.36, 95% CI [0.20, 0.53], *p* < .001), adventurousness-confidence (*b* = 0.32, 95% CI [0.18, 0.46], *p* < .001), and intelligence (*b* = 0.16, 95% CI [0.01, 0.32], *p* = .04) and a lower on the dimension vitality-attractiveness (*b* = −0.29, 95% CI [−0.43, −0.14], *p* < .001). We found no significant interactions between timepoint and participants’ sex (Supplemental Tables S10 and S11).

Finally, we explored mean-level changes separately in the group of participants who entered one relationship lasting longer than 6 months over the investigated time span and in the group of participants who entered more than one relationship lasting longer than 6 months. The number of participants who did not enter any relationship lasting longer than 6 months was fairly small, hence we refrain from interpreting these results. Because these exploratory analyses were not preregistered, we take a more conservative approach and only interpret findings with a *p* < .01. For those participants who had entered more than one relationship and mirroring the results of our main analyses, we found the same significantly increased preferences for the warmth-trustworthiness (*M*_T1_ = 4.12, *SD*_T1_ = 0.48, *M*_T2_ = 4.31, *SD*_T2_ = 0.48, *p* = .001, *d* = 0.40 [0.16, 0.65]) and status-resources (*M*_T1_ = 2.74, *SD*_T1_ = 0.60, *M*_T2_ = 2.94, *SD*_T1_ = 0.61, *p* = .007, *d* = 0.32 [0.09, 0.55]) as for the entire sample. Participants who had entered one relationship did not show the previously found significant increase in status-resources (*M*_T1_ = 2.73, *SD*_T1_ = 0.61, *M*_T2_ = 2.86, *SD*_T2_ = 0.61, *p* = .050, *d* = 0.22 [0.00, 0.44]) and the increase in warmth-trustworthiness (*M*_T1_ = 4.18, *SD*_T1_ = 0.43, *M*_T2_ = 4.22, *SD*_T2_ = 0.68, *p* = .633, *d* = 0.05 [−0.19, 0.31]; see Supplemental Material S2I).

### Age Effects

**Figure 1.**
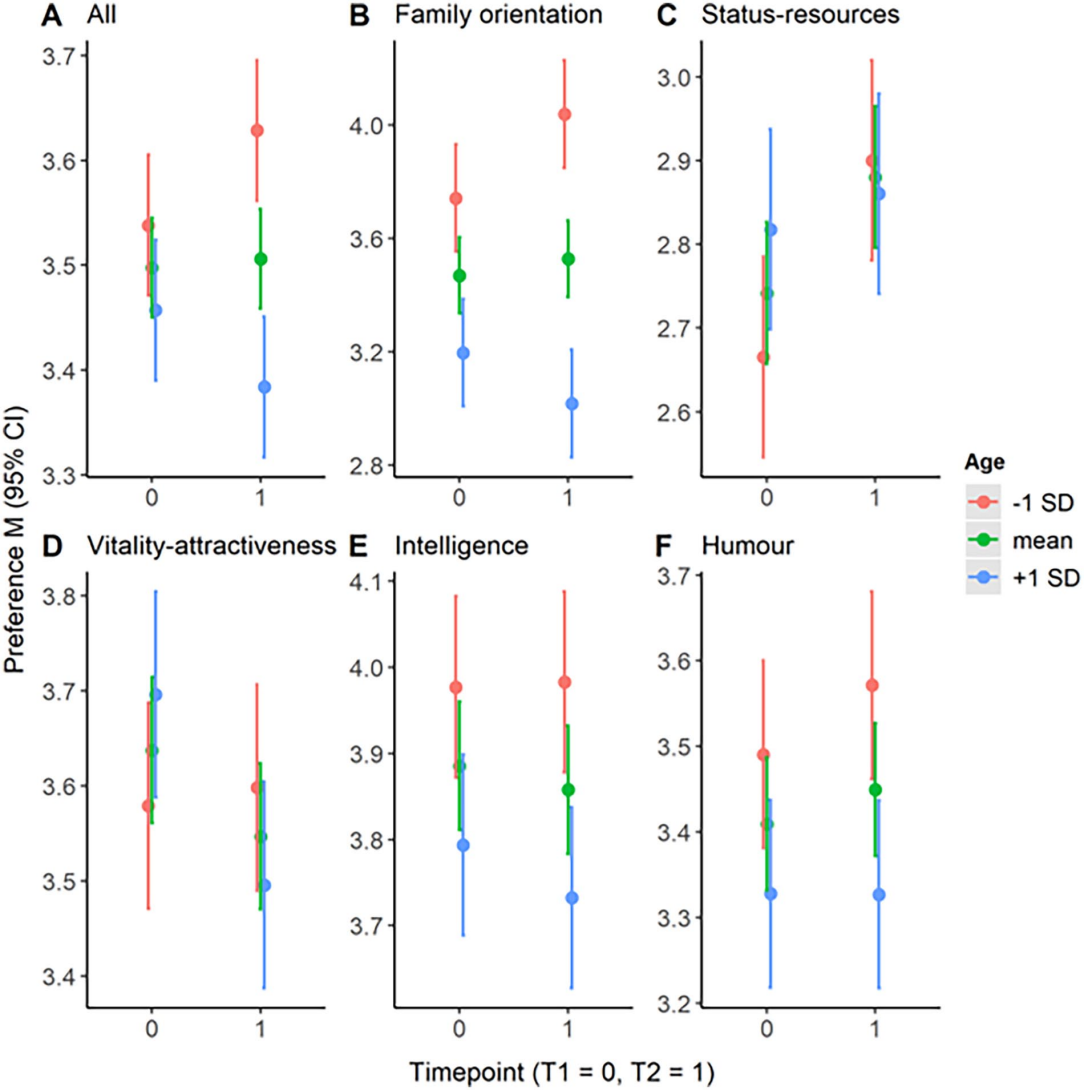
Participant’s Mean Ideal Partner Preferences at T1 and T2 Divided Into Different Age Groups. *Note.* On the *y*-axis, participant’s mean preference (95% CI) across all dimensions (A), and separately for the dimension family orientation (B), status-resources (C), vitality-attractiveness (D), intelligence (E), and humor (F) plotted separately for each time point (0 = T1, 1 = T2) on the x-axis. To facilitate understanding of our plot, participants were divided into three age groups for illustration (red = *Mean* age −1 *SD*, green = *Mean* age, blue = *Mean* age +1 *SD*). However, we analyzed age continuously. Note that the y-axis differs between each plot. CI = confidence interval.

We investigated the relationship between age and mean-level changes across all dimensions using multilevel models as we predicted increases from T1 to T2 in the dimension status-resources (H2.1) and family-orientation (H3.1), especially when participants had been younger at T1. We predicted participants’ preferences with the time point (0 = T1, 1 = T2), age (*z*-standardized), and their interaction while including a random effect for participants because of the repeated measurement. For status-resources, we found a significant main effect of time point and interaction of participants’ age and time point (Table 4). More specifically, the previously described increase over time in status-resources preferences replicated, yet, as predicted in H2.1, the significant interaction suggested a stronger increase over time for younger participants (Figure 1C, red vs. blue line).

**Table 4.** Multilevel Models Investigating the Association of Age on Each Dimension.

Predictors	Warmth-trustworthiness		Vitality-attractiveness		Status-resources		Family orientation	
	*b* [95% CI]	*p*	*b* [95% CI]	*p*	*b* [95% CI]	*p*	*b* [95% CI]	*p*
Time point	0.09 [0.01, 0.18]	.034	−0.09 [−0.17, −0.01]	.027	0.14 [0.05, 0.22]	.002	0.06 [−0.09, 0.21]	.442
Age at T1	−0.02 [−0.10, 0.05]	.527	0.06 [−0.02, 0.14]	.135	0.08 [−0.01, 0.16]	.078	−0.27 [−0.41, −0.14]	<.001
Time point × age	−0.05 [−0.14, 0.03]	.243	−0.11 [−0.19, −0.03]	.007	−0.10 [−0.18, −0.01]	.029	−0.24 [−0.39, −0.09]	.002
Predictors	Intelligence		Creativity		Humor		Adventurousness−confidence	
	*b* [95% CI]	*p*	*b* [95% CI]	*p*	*b* [95% CI]	*p*	*b* [95% CI]	*p*
Time point	−0.03 [−0.11, 0.06]	.520	−0.09 [−0.18, 0.01]	.070	0.04 [−0.05, 0.13]	.365	−0.06 [−0.13, 0.02]	.140
Age at T1	−0.09 [−0.17, −0.02]	.016	0.03 [−0.06, 0.11]	.542	−0.08 [−0.16, −0.00]	.040	−0.02 [−0.09, 0.06]	.645
Time point × age	−0.03 [−0.12, 0.05]	.436	−0.02 [−0.12, 0.07]	.604	−0.04 [−0.13, 0.05]	.356	−0.06 [−0.13, 0.01]	.113

*Note.* Full models can be found in our supplement (Supplemental Material S2D). CI = confidence interval.

For the dimension family orientation, we found a significant main effect of age and its interaction with time point. More specifically, and contradicting H3, we did not find an overall increase in preference for family orientation, but as predicted in H3.1, preferences for family orientation increased from T1 to T2 for younger participants. Interestingly, younger compared with older participants already had a higher preference for T1 (Figure 1B).

Exploratorily, we investigated the association of age with all other dimensions. Preferences for vitality-attractiveness decreased, especially for older participants (Figure 1D). Furthermore, significant main effects of age for intelligence and humor suggested decreased preferences for both dimensions when being older (Figure 1E, F). When only interpreting results with *p* values <.01, only the interaction of age and time point remained significant, but the main effect for vitality-attractiveness did not.

### Participants With Versus Without Children

As we predicted that the desire for status-resources changes with the immediacy of a desire for or actual existence of children (H4), we also investigated the association of parenthood and participants’ preferences. After excluding participants who already had children at T1 (*n* = 34, 16%), we divided our sample into two groups: *n* = 63 participants (37%) without children or the intention to have a child and *n* = 107 participants (63%) who have had at least one child since T1 or the intention to have a child (Supplemental Table S2), henceforth referred to as participants with versus without children.

We correlated T1 and T2 preferences across all dimensions separately for each group and compared correlation coefficients using a two-sided *z*-test. Overall, mean rank-order stability was lower in participants with, as compared to without children (*r_with_* = .32 vs. *r_without_* = .54, *p* = .036). Table 5 depicts rank-order stabilities for each dimension. As predicted in H4, rank-order stability for status-resources was lower in participants with, as compared to without children. Their rank-order stability was also lower for warmth-trustworthiness, intelligence, creativity, and humor but not in the remaining three dimensions.

**Table 5.** Means, Standard Deviations, Effect Sizes, and Correlations of T1 and T2 Importance Ratings Separately for Participants With and Without Children.

	With children (*n* =107)					Without children (*n* = 63)					p_comparison_
Preference dimension	*M_T1 with_* (SD_T1 with_)	*M_T2 with_* (SD_T1 with_)	*p_with_*	*d_with_* [95% CI]	*r_with_*	*M_T1 without_* (*SD*_T1 without_)	*M_T2 without_* (*SD*_T2 without_)	*p_without_*	*d_without_* [95% CI]	*r_without_*	
Warmth-trustworthiness	4.19 (0.43)	4.35 (0.58)	.011	0.31 [0.07, 0.55]	.25	4.08 (0.53)	4.19 (0.52)	.098	0.20 [−0.04, 0.43]	.57	.001
Vitality-attractiveness	3.64 (0.55)	3.55 (0.61)	.106	−0.16 [−0.35, 0.04]	.47	3.61 (0.53)	3.59 (0.52)	.703	−0.04 [-0.28, 0.19]	.57	.190
Status-resources	2.68 (0.64)	2.87 (0.62)	.007	0.30 [0.08, 0.52]	.37	2.74 (0.58)	2.83 (0.63)	.185	0.15 [−0.07, 0.38]	.60	.006
Family orientation	3.85 (0.72)	4.23 (0.74)	<.001	0.52 [0.25, 0.79]	.14	2.89 (1.05)	2.58 (1.09)	.055	−0.29 [−0.59, 0.01]	.30	.285
Intelligence	3.94 (0.51)	3.90 (0.58)	.556	−0.07 [−0.30, 0.16]	.28	3.85 (0.56)	3.88 (0.55)	.686	0.05 [−0.19, 0.28]	.57	.005
Creativity	3.50 (0.57)	3.42 (0.68)	.221	−0.14 [−0.36, 0.08]	.34	3.50 (0.59)	3.48 (0.59)	.726	−0.04 [−0.27, 0.19]	.59	.006
Humor	3.53 (0.48)	3.53 (0.59)	.960	−0.01 [−0.23, 0.22]	.28	3.28 (0.53)	3.41 (0.62)	.080	0.22 [−0.03, 0.47]	.52	.023
Adventurousness-confidence	3.18 (0.51)	3.13 (0.56)	.367	−0.09 [−0.29, 0.11]	.43	3.12 (0.53)	3.08 (0.58)	.553	−0.07 [−0.31, 0.17]	.54	.250

*Note.* The lowercase “with” refers to the group of participants with children, the lowercase “without” refers to participants without children. To show how preferences developed for each group, mean level changes are displayed. However, only results of multilevel models are interpreted for these exploratory analyses. *z*-transformed correlation coefficients of each group are compared using a z-test with the column *p_comparison_* referring to the *p*-values of each comparison. CI = confidence interval.

As a robustness check, we repeated our analyses but divided our sample into participants with and participants without children, not taking into account participants’ intention to have a child. Our results remained virtually identical (Supplemental Table S8). These results suggest that having children may be associated with altered partner preferences.

**Figure 2.**
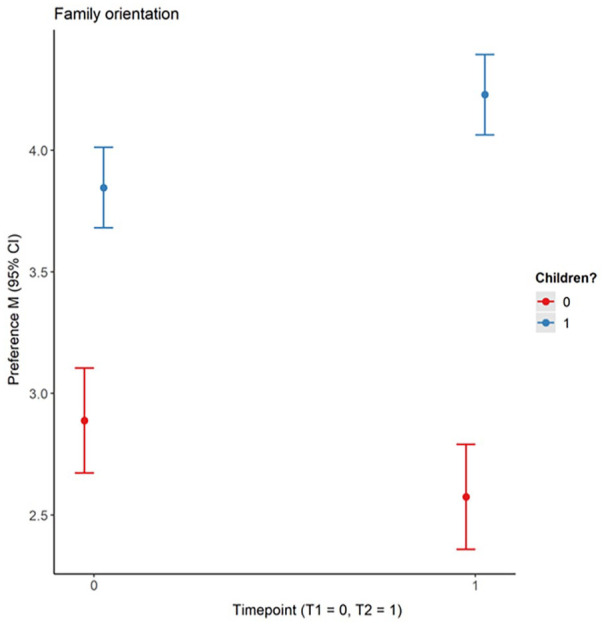
Preference for Family Orientation at T1 and T2 for Participants With Versus Without Children. *Note.* The dots depict participants’ mean preference for family orientation at each time point (0 = T1, 1 = T2). The lines crossing each dot display the 95% CI. In red, participants without children (*n* = 63, 37%) and in blue participants who have had at least one child since T1 (*n* = 107, 63%). 34 (16%) participants who already had a child at T1 were not included in these analyses. The figure shows that participants’ preference for family orientation increases from T1 to T2, but only in the group of participants with children. Participants without children place less importance on family orientation which does not change significantly over time. CI = confidence interval.

Exploratorily, we investigated mean-level changes on the level of dimensions and across all items by running multilevel models as the previous analyses focusing on rank-order coefficients investigated the stability of preferences in relation to having children but not the direction of potential changes in relation to having children. We predicted ideals with the time point (0 = T1, 1 = T2), whether participants have children (0 = *without children*, 1 = *with children*), their interaction, and a random effect taking the repeated measurement into account (Supplemental Material S2E). Overall, participants with children reported higher ideals (*b* = 0.18, 95% CI [0.07, 0.29], *p* < .001). Analyses on the level of dimensions revealed that participants with children had a significantly higher preference for the dimension humor (*b* = 0.25, 95% CI [0.08, 0.43], *p* = .004). We did not find further significant effects except for family orientation (time point: *b* = −0.31, 95 % CI [−0.58, −0.05], *p* = .023; having a child: *b* = 0.96, 95% CI [0.69, 1.23], *p* < .001; their interaction: *b* = 0.70, 95% CI [0.36, 1.03], *p* < .001), suggesting that the importance of family orientation increased over time but only for participants with children. Participants without children placed less importance on this dimension at both assessments (Figure 2).

### Insight Into Changes in Ideal Partner Preferences

Finally, we predicted that peoples’ perception of change may correspond to their actual changes in preferences for the dimensions status-resources (H5.1) and vitality-attractiveness (H5.2). Therefore, we investigated whether participants have insight into how their preferences have changed. First, we subtracted participants’ T1 from their T2 preferences for each dimension, indicating the actual change in preferences. Descriptively, participants perceived themselves to place less importance on the dimensions vitality-attractiveness, status-resources, creativity, and adventurousness-confidence, and increased importance on the dimensions warmth-trustworthiness, family orientation, intelligence, and humor over time. Importantly, for all dimensions except family orientation, about half of the sample did not perceive themselves to have changed (Table 6). Second, to estimate whether participants have insight into their actual changes, we correlated their perceived change with their actual change for each preference dimension. We found a mean accuracy correlation of *r* = .20. Across dimensions, coefficients ranged from *r* = .09 to *r* = .45, suggesting considerable variation in how much insight people had into how much their preferences had changed since T1. For example, as shown in Table 6, participants were quite accurate regarding their change in preference for family-orientation but had almost no insight into their changes in preference for intelligence. In contrast to H5.1, participants did not show any insight regarding their preference change for status-resources: Preferences increased over time, but participants perceived to have ascribed less importance to this dimension. In line with H5.2, participants accurately perceived a decreased preference for vitality-attractiveness over time. We found no interindividual differences in participants’ insight into how their preferences have changed when investigating the effects of age or sex and their respective interaction (S2F).

**Table 6.** Participants’ Actual and Perceived Change of Each Preference Dimension and Their Correlations.

	Actual change	Perceived change	Responses of perceived change (%)					Insight	
	*M* (*SD*)	*M* (*SD*)	−2	−1	0	1	2	*r* [95% CI]	*p*
Warmth-trustworthiness	0.09 (0.62)	0.43 (0.72)	0.0	3.4	59.8	27.0	9.8	.20 [0.06, 0.33]	.005
Vitality-attractiveness	−0.09 (0.59)	−0.27 (0.72)	2.9	32.8	54.4	8.3	1.5	.21 [0.07, 0.33]	.003
Status-resources	0.14 (0.63)	−0.11 (0.82)	4.9	22.6	52.9	17.7	2.0	.09 [−0.04, 0.23]	.178
Family orientation	0.06 (1.11)	0.08 (1.20)	12.8	15.2	37.3	20.6	14.2	.45 [0.34, 0.56]	<.001
Intelligence	−0.03 (0.62)	0.21 (0.62)	1.0	3.4	73.5	17.7	4.4	.09 [−0.05, 0.22]	.208
Creativity	−0.09 (0.69)	−0.12 (0.75)	2.5	25.5	54.9	15.7	1.5	.24 [0.10, 0.36]	.001
Humor	0.04 (0.63)	0.05 (0.63)	1.0	12.3	69.6	15.2	2.0	.14 [0.01, 0.28]	.039
Adventurousness-confidence	−0.06 (0.54)	−0.16 (0.80)	1.5	33.3	48.5	13.2	3.4	.18 [0.05, 0.31]	.009

*Note.* Negative values indicate a decreased importance and positive values indicate an increased importance of each corresponding preference dimension. *r* [95% CI] = correlation coefficient and 95% confidence interval between actual and perceived changes. *p* = *p*-values of correlation coefficients. CI = confidence interval.

## Discussion

In this study, employing unique longitudinal data across 13 years, we investigated stability (i.e., retest and profile correlations) and change (i.e., mean-level changes) of ideal partner preferences, and whether individuals possess insight into how their preferences have changed (i.e., correlations of perceived changes with actual changes).

### Stability and Change in Ideal Partner Preferences

Regarding our first hypothesis (H1), our results suggested considerable overall stability of participants’ ideals of *r* = .42, corresponding to a medium-sized to large effect (Cohen, 2013; Gignac & Szodorai, 2016). This stability is smaller than coefficients obtained after 5 months (Gerlach et al., 2019) yet roughly comparable to coefficients found after 3 years (Bleske-Rechek & Ryan, 2015). These results are in a range that has previously been reported for the rank-order stability of personality traits (around *r* = .60 for a retest interval of 6.7 years, Roberts & DelVecchio, 2000; *r* = .33 for an interval of 11 years in a more diverse sample, Atherton et al., 2022). However, when compared with a meta-analysis by Anusic and Schimmack (2016), our results are comparable with the retest correlation of affect and self-esteem found after 13 years in a group of 30-year-olds but smaller compared to the retest correlation of broad personality dimensions after 13 years in a group of 30-year-olds. Our results, together with the stability coefficients reported for partner preferences across shorter intervals (e.g., (Fletcher et al., 1999, 2000; Gerlach et al., 2019; Shackelford et al., 2005), are in line with the finding that the strongest declines in stability coefficients are found in the first years after assessment (Anusic & Schimmack, 2016; Costa et al., 2019). The fact that our retest correlations do not further decrease even over such a long timespan suggests that individual differences in ideal partner preferences contain a sizable trait component (Anusic & Schimmack, 2016). However, this stability seems to be more comparable to constructs such as self-esteem (as opposed to broader personality domains), which has been shown to be more susceptible to external influences.

Nonetheless, investigating participants’ profiles revealed that patterns of which traits individuals preferred more or less were surprisingly stable, with overall profile correlations exceeding *r* = .70. These profile stabilities were only slightly reduced when accounting for normative components (e.g., most people value warmth-trustworthiness more than status-resources) by employing distinct profile correlations. We take this high temporal consistency to suggest that—while individual dimensions may well be affected by external influences, resulting in only moderate stability—idiosyncratic patterns in what people value in a romantic partner may be a very stable individual difference characteristic, even when effects of what is normatively more versus less preferred are taken into account.

We then examined the relationship between parenthood and the stability of preferences. As put forward in hypothesis four (H4), we found that the stability of preferences for status-resources was lower in participants who became parents over the 13-year study period or who had intentions to become a parent at the time of the re-assessment, compared with participants without (the intention to have) children. We assumed that these shifts in partner preferences could be related to shifting priorities and efforts according to different life stages (cf. Del Giudice et al., 2016; Heckhausen et al., 2010, 2019), with parenthood potentially being of particular importance. As having a partner who is able to provide resources facilitates founding a family and raising children, (the decision to) becoming a parent may alter one’s preference for status-resources, explaining the lower stability. Yet, parenthood was also related to the stability of other preference dimensions, suggesting that the decision to become a parent has the potential to shake up how we picture our ideal partner more generally.

We also investigated mean-level changes in ideal partner preferences. In line with our second hypothesis (H2), participants placed higher importance on status-resources over time and this increasing preference was stronger for younger participants. Furthermore, although effects were small (Cohen’s *d* < 0.20), participants placed more importance on warmth-trustworthiness and less on vitality-attractiveness over time. Our third prediction (H3), an increase in family-orientation, was only partly supported: Over time, the preference for family-orientation only increased for younger individuals, yet compared with older participants, younger individuals already reported a higher preference for family-orientation at the initial assessment. Further exploration revealed that participants without children generally placed less importance on family-orientation, whereas the preference for family-orientation increased over time for those with children. While this might be a mere cohort effect, this finding could also be interpreted in light of age-graded opportunity structures and/or developmental deadlines (Wrosch & Heckhausen, 2005). For example, younger participants might picture themselves as likely to begin a family in the future, whereas older participants had already begun to ponder a possible life without children because they already considered themselves to be beyond the ideal age for having children, were pessimistic about finding a suitable partner for such an endeavor, or had already come to cherish a “childfree” lifestyle. Exploring mean-level changes in relation to the number of relationships participants had entered revealed that participants who entered more than one relationship over time reported an increased preference for warmth-trustworthiness and status-resources, whereas participants who entered only one relationship over time showed no significant increase in these preference dimensions. For the dimension status-resources, this may be due to having limited statistical power in these analyses, as participants who entered only one relationship descriptively showed an increased preference for status-resources. It can also be speculated that participants who entered more than one committed relationship after going through one or more break-ups may have realized that having a warm and trustworthy partner may be most vital for a relationship to last. Participants who entered only one committed relationship, however, may not have seen the necessity to update their preferences on this dimension.

In our study, we found considerable stability of preferences over 13 years. As such, our findings cannot explain the mixed findings in previous research on the link between preferences and relationship decisions. An alternative explanation for those mixed findings may be the relationship phase investigated (see Campbell & Stanton, 2014; Gerlach et al., 2019): studies that could not find a link between preferences and relationship decisions, for the most part, investigated the initial stage of getting to know each other (e.g., Eastwick & Finkel, 2008; Joel et al., 2017; Todd et al., 2007), whereas studies finding a relationship between preferences and relationship decisions often investigated already established relationships (e.g., Conroy-Beam & Buss, 2016; Park & MacDonald, 2019) or relationship formation (e.g., Campbell et al., 2016; Gerlach et al., 2019). However, our findings have important implications for future research investigating the association of ideal partner preferences and relationship decisions. First, the relatively high stability of preferences suggests that studies which investigate the association between preferences and relationship decisions do not necessarily need to constantly assess preferences over the investigated timespan. Instead, study designs in which preferences are initially assessed should suffice to investigate the link between these initial preferences and later relationship decisions. Second, a factor to consider when investigating longer timespans or populations more diverse as the typical student sample is that having children may alter preferences. Future studies investigating partner preferences may thus take into account the parenthood status of participants and the presence (vs. absence) of family formation goals more broadly.

### Insight Into Preference Change

Over the 13-year study period, preferences for status-resources and warmth-trustworthiness increased and decreased for vitality-attractiveness—but were these changes mirrored in participants’ perceptions? Descriptively, participants perceived increases in their preference for warmth-trustworthiness and perceived decreases in their preference for vitality-attractiveness and status-resources. They also perceived increases in family orientation, intelligence, and humor and decreases in adventurousness-confidence and creativity. One interpretation of these perceived changes may be that participants believe to place more importance on dimensions that become more relevant with increasing age. For example, with increasing age, it may be adaptive to have a partner who is caring and oriented toward the family instead of a partner who is up for adventure and likes taking risks. Although objectively, having a high status and resources might also become more important when one gets older, participants may not perceive this change because they might have already achieved certain resources or status for themselves and may not realize that this increased standard of living has already shaped their preferences for a partner. Another possibility is that participants may answer in a socially desirable way: If participants do not want to admit that having a certain status and monetary resources is relevant to them, they might indicate that this dimension had become less relevant to them over time, while still ascribing considerable importance to it.

Interestingly, around 50% of participants did not report that they changed their ideals, except for family orientation, where only 37% of participants believed that their preferences had not changed. These patterns dovetail with results by Sprecher and colleagues (2018): Around half of their sample perceived not to have changed their ideals, except for “good parenting potential,” a variable close to family orientation. This perception of no change may mirror the previously found stability of ideal partner preferences or changes may have occurred at a younger age (Bleske-Rechek & Ryan, 2015).

When investigating whether perceptions correspond to actual changes, overall, we found a small positive effect. Yet, insight varied considerably between the different dimensions: Participants had the most insight into family orientation and the least for status-resources and intelligence. Contradicting our fifth hypothesis (insight into changes for status-resources, H5.1), participants believed to have decreased in their preference, when in fact they increased over time. One possibility is that participants may perceive themselves in a biased self-enhancing manner via a similar process to what Robins et al. (2005) suggested to be the case for perceived changes in personality. Yet, in line with the second part of this prediction (H5.2), participants showed some insight into changes in their preference for vitality-attractiveness, although the perception of change appears stronger than the actual change. Interestingly, age and sex were not related to participants’ insight.

The present results for perceptions are in line with previous research (Bleske-Rechek et al., 2009) that found participants to predict that they would value intrinsic characteristics (i.e., warmth-trustworthiness, family orientation) more and appearance (i.e., vitality-attractiveness) less over time, suggesting that participants may be more oriented toward committed relationships over time. At the same time, perceptions of change were somewhat exaggerated and for the most part only achieved modest accuracy (family orientation being a notable exception), showing that perceptions do not necessarily correspond to actual changes. These results highlight the necessity to conduct longitudinal studies when one is interested in preference change and underscore that intraindividual processes should not be investigated in cross-sectional data: Self-perceptions of change do not reflect actual changes accurately enough to allow them to be used as a substitute.

### Strengths

The longitudinal design of this study, covering 13 years, makes it unique among studies on the stability and change of partner preferences, which have so far investigated much shorter time periods. Even over this long timespan, we managed to rerecruit a sizable proportion of the initial sample, and participant retention was better than expected over such a large time interval. For example, while in the current study we found a retention rate of 59% after 13 years, Gerlach et al. (2019) reported a retention rate of more than five months of 64%, whereas a study by Gustavson et al. (2012) covering a time span of 15 years reported a retention rate of 44%. A special feature of our sample is that it is a community sample not restricted to the typical student population. In particular, our sample spanned a wide age range, allowing us to investigate intraindividual stability and change of preferences across a period when participants were still single until much later in life when they may have found a partner with whom they then had to decide whether to have children or not. Investigating this life stage may be of particular interest since it does not only involve the time in which participants start having a family but also a time in which important career decisions take place. Finally, we used comprehensive measures of participants’ ideals at both assessments and complementary indices to investigate their stability and change.

### Limitations and Future Directions

Although our community sample was arguably more diverse than the typical student sample, it was still highly educated and came from a Western background. The generalizability of our results may be limited because preferences and their importance could not only vary by education but also across different cultures. For example, in a study involving samples from Taiwan, Lam et al. (2016) uncovered preference attributes referring to the extended family previously overlooked in Western samples. Furthermore, education might be related to how much importance individuals ascribe to attributes conducive to a partner’s career advancement (e.g., successful, ambitious). Future studies should strive to recruit participants with more diverse educational backgrounds, ideally also from non-Western countries (Henrich et al., 2010).

Furthermore, although the large retest period is unique and showed that ideal partner preferences contain a sizable trait component, life events may still be associated with a change in preferences. The fact that we only had two assessments available precludes an in-depth analysis of further factors that might have driven preference change. Future research should include multiple assessments of preferences and important events (e.g., parenthood; entering Gerlach et al., 2019 or ending relationships; experiences of romantic rejection and acceptance Charlot et al., 2020). Additional factors influencing changes in partner preferences may be the increased occurrence of specific life events in a persons’ social environment. For example, the importance of having a partner with a high family orientation may increase when more and more people in one’s environment are trying to or are indeed having a child (Keim et al., 2009). Another possible change in partner preferences may be that people lower their expectations after a long period of time not being able to find a partner (Gerlach et al., 2019). For example, people lower their standards regarding a partner’s physical attractiveness. Finally, a recent study has found divorce to be associated with changes in self-esteem (Bleidorn et al., 2021). Similarly, relationship dissolution may be a life event associated with changes in preferences. For example, after a relationship dissolution fraught with conflict, individuals may increase their preference for having a kind, trustworthy partner because they recently got to know the disagreeable side of their ex-partner. Future research with multiple assessments should also include participants’ perception of change to investigate what drives the accuracy of preference change perceptions and whether the perception of change may be associated with future dating or relationship decisions.

Finally, we deviated from our preregistered analytic plan in three analytic decisions (see S4). Therefore, only our hypotheses and design can be regarded as preregistered. In particular, the diverging assessment of initial ideals between the two samples led to larger problems than anticipated, which led us to the decision to analyze both samples separately and interpret results based on the BSDS only. Unfortunately, this also lowered our sample size, hence the power of our study, which is especially relevant for the analyses comparing participants with and without children. Furthermore, as the instruction for rating partner preferences was not completely identical across T1 and T2, we also checked for measurement invariance across the two time points according to the procedure as suggested by Mackinnon et al. (2022). We found scalar invariance partly supported, suggesting that participants may have interpreted our response scale slightly differently at T1 and T2. We therefore recommend future studies to adopt the exact same wording of their instructions at all assessments.

## Conclusion

We provide evidence that ideal partner preferences are considerably stable over 13 years, with some changes being related to life events such as parenthood. The importance of a partner with status and resources increased over time, with this increase being stronger for younger individuals. For some preferences (e.g., family orientation), participants knew how they had changed over time, while for other preferences change perceptions did not mirror actual changes. Future research should investigate further factors influencing stability and change in ideals as well as the factors facilitating or hindering insight into such changes.

## Supplemental Material

sj-docx-1-psp-10.1177_01461672231164757 – Supplemental material for Stability and Change of Individual Differences in Ideal Partner Preferences Over 13 Years Supplemental material, sj-docx-1-psp-10.1177_01461672231164757 for Stability and Change of Individual Differences in Ideal Partner Preferences Over 13 Years by Julie C. Driebe, Julia Stern, Lars Penke and Tanja M. Gerlach in Personality and Social Psychology Bulletin

## Notes

1. We calculated mean–retest correlations by calculating the mean of Fisher *z*-transformed correlation coefficients, as they were originally reported by Fletcher and colleagues (1999, 2000), Bredow and Hames (2019), and Shackelford and colleagues (2005a).

2. Fletcher et al. (1999) investigated the stability of preferences in a sample of 42 participants (25 women and 17 men) with a mean age of 23.0 years (*SD* = 4.7). In this sample, 22 participants were single and 20 participants were dating, married, or lived together. Fletcher et al. (2000) investigated a sample of 100 participants (65 women and 35 men) with a mean age of 20.9 years (*SD* = 4.60). All of these participants were in a romantic relationship. Gerlach et al. (2019) investigated a sample of 763 participants (519 women and 244 men) with a mean age of 25.0 years (*SD* = 4.8). While in this sample, everyone was single at the first assessment, 258 participants were involved in some sort of a romantic relationship 5 months later. Bredow and Hames (2019) investigated a sample of 285 participants (205 women and 79 men) with a mean age of 30.5 years (*SD* = 10.8). At T1, 52.6% were involved in a romantic relationship. Shackelford et al. (2005a) investigated a sample of 27 married couples with women having a mean age of 25.5 years (*SD* = 4.1) and men having a mean age of 26.8 years (*SD* = 3.8). Bleske-Rechek and Ryan (2015) investigated a sample of 200 participants (143 women and 57 men). At T2, participants had a mean age of 21.5 years (*SD* = 1.24). The authors did not report participants’ relationship status.

3. Because, so far, previous research did not investigate participants’ actual insight into how their preferences have changed, it was difficult to formulate specific hypotheses. However, results of an unpublished thesis from our lab indicated that participants of a different study had the most insight into the dimensions status-resources and vitality-attractiveness. Hence, we decided to formulate hypotheses for these two dimensions only and investigate all other dimensions in a more explanatory manner.

4. This bonus was disbursed if participants invited their peers and partners to a second part of the data collection.

5. At T1, this sample consisted of *N* = 142 participants, of which *n* = 66 individuals could be re-recruited for a T2 reassessment.

6. Aside from the small sample size, this second sample came with the limitation of a far less extensive assessment of ideal partner preferences. Only 13 items assessed ideal partner preferences and each entailed several characteristics, for example, “parental abilities, wish for children.” These items and response formats differed from the BSDS.

7. At T2, participants rated 72 items and 59 of these items at T1. Thus, we excluded 14 items: 13 items because of missing values at T1 and one item because it did not load on any factor.
