# Supplementary material for: Stability and Change of Individual Differences in Ideal Partner Preferences Over 13 Years
Source: Pers Soc Psychol Bull. 2023 Apr 8;50(8):1263–79. doi: 10.1177/01461672231164757 (PMC11193321; doi:10.1177/01461672231164757)
Supplement: sj-docx-1-psp-10.1177_01461672231164757 – Supplemental material for Stability and Change of Individual Differences in Ideal Partner Preferences Over 13 Years [file sj-docx-1-psp-10.1177_01461672231164757.docx]

**Supplement**

**Stability and Change of Individual Differences in Ideal Partner Preferences over 13 Years**

[S1. 3](#_Toc126572754)

[A. Further Descriptives 3](#_Toc126572755)

[B. Descriptive Data of Participants of the Sociosexuality Study 7](#_Toc126572756)

[C. Preference Dimensions 9](#_Toc126572757)

[D. Measurement Invariance 11](#_Toc126572758)

[S2. Robustness Checks 13](#_Toc126572759)

[A. Rank-order Stability 13](#_Toc126572760)

[B. Profile Stability 16](#_Toc126572761)

[C. Relationship of Sex with Mean-level Changes 17](#_Toc126572762)

[D. Relationship of Age with Mean-level Changes 19](#_Toc126572763)

[E. Relationship of Parenthood and Mean-level Changes 21](#_Toc126572764)

[F. Insight into Preference Change 23](#_Toc126572765)

[G. Relationship with Age 24](#_Toc126572766)

[H. Relationship with Sex 25](#_Toc126572767)

[I. Relationship with Relationship Status 26](#_Toc126572768)

[J. Relationship with Relationship Length 30](#_Toc126572769)

[S3. Results for each study and across studies 32](#_Toc126572770)

[A. Rank-Order Stability 32](#_Toc126572771)

[B. Profile Correlation 33](#_Toc126572772)

[C. Mean-level Changes 34](#_Toc126572773)

[D. Association of Age 36](#_Toc126572774)

[E. Association of Parenthood 40](#_Toc126572775)

[F. Insight into Preference Change 42](#_Toc126572776)

[S4. Deviations to Our Preregistration 45](#_Toc126572777)

[S6. Conference Presentation 46](#_Toc126572778)

[S7. Author Contributions 46](#_Toc126572779)

[S8. References 47](#_Toc126572780)

#

# **S1.**

## **A.** **Further Descriptives**

**Figure S1**

Illustration of our study structure


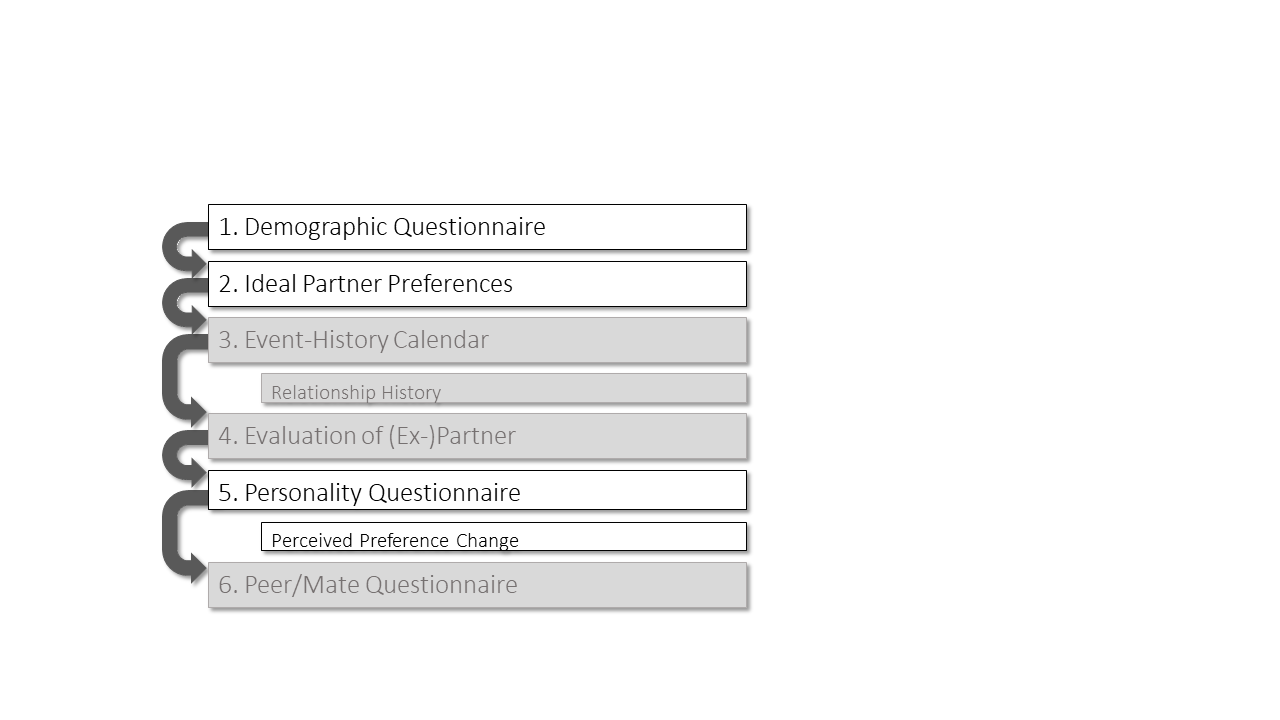


*Note.* Steps highlighted in light grey are of minor importance for the current study.

**Figure S2**

*Participant flow
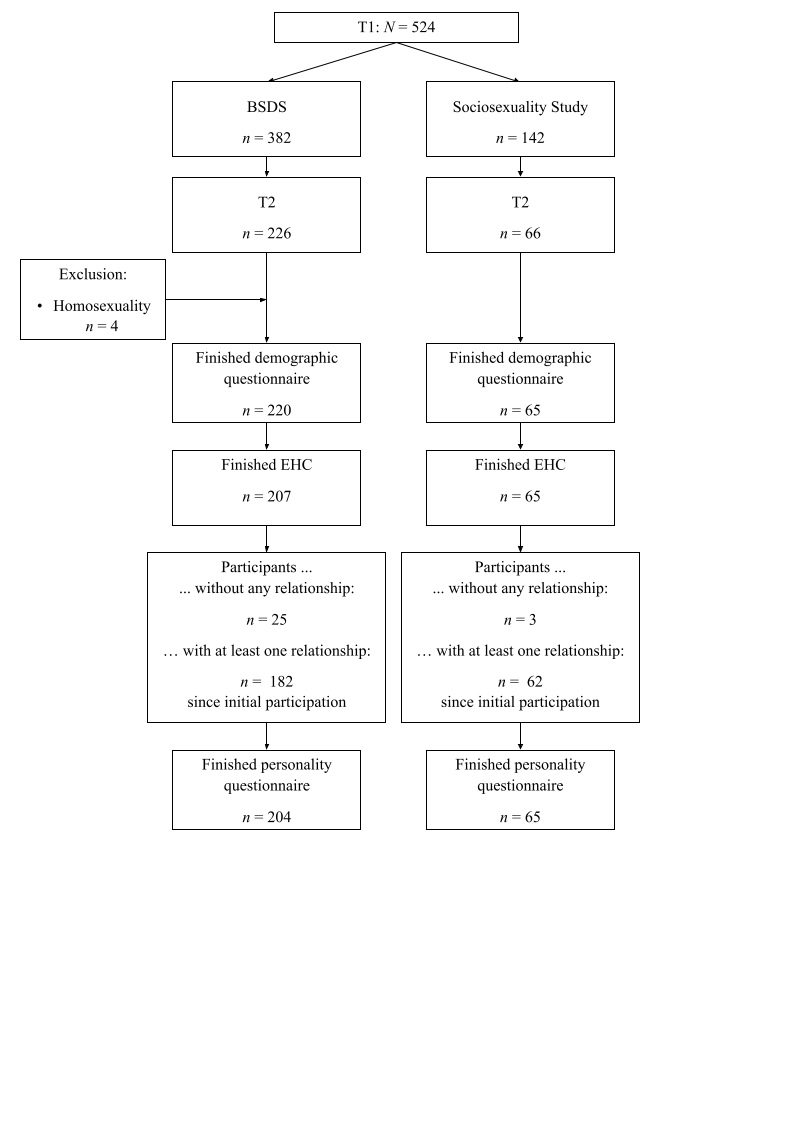
*

**Table S1**

*Attrition analysis comparing group differences between participants who dropped out from T1 to T2 and between participants who dropped out during T2*

| Study | T1 values | | | T2 values | | |
| --- | --- | --- | --- | --- | --- | --- |
| Participation | Dropouts T1 | Re-recruited |  | Dropouts T2 | Final Sample |  |
| *n* | 156 | 226 |  | 22 | 204 |  |
| Variable | *M* (*SD*) | *M* (*SD*) | *p* | *M* (*SD*) | *M* (*SD*) | *p* |
| Age | 33.06 (7.80) | 32.56 (7.05) | .53 | **41.86 (6.96)** | **46.19 (7.35)** | **.01** |
| Sex. orientation | 1.09 (.29) | 1.13 (.36) | .25 | 1.77 (1.66) | 1.08 (.31) | .07 |
| O | 3.82 (.53) | 3.83 (.50) | .87 | 3.68 (.89) | 3.91 (.58) | .56 |
| C | **3.89 (.60)** | **3.77 (.62)** | **.05** | 3.07 (.69) | 3.63 (.62) | .14 |
| E | 3.57 (.52) | 3.47 (.06) | .11 | 3.65 (.73) | 3.42 (.76) | .52 |
| A | 3.81 (.50) | 3.81 (.54) | .99 | 3.02 (.99) | 3.53 (.60) | .32 |
| N | **2.52 (.70)** | **2.69 (.72)** | **.03** | 3.23 (1.18) | 2.86 (.85) | .53 |

*Note. p*-values are reported for the analysed mean-level changes using *t*-tests (two-sided). Dropouts T1 = group of participants who did not participate at T2; Re-recruited = group of participants who participate at T2; Dropouts T2 = group of participants who had to be excluded or dropped out during T2; Final Sample = final sample at T2; O = Openness; C = Conscientiousness; E = Extraversion; A = Agreeableness; N = Neuroticism; Being assessed using the BFI (Lang, Luedtke, & Asendorpf, 2001) at T2 and the NEO-FFI (Borkenau & Ostendorf, 1993) at T1. Boldface type indicates significant differences with the threshold at the .05.

**Table S2**

*Overview of participants with and without children.*

| Variable | Sample at T2 | | Women | | Men | |
| --- | --- | --- | --- | --- | --- | --- |
|  | Yes *n* | No *n* | Yes *n* | No *n* | Yes *n* | No *n* |
| Child at T1 | 34  (16%) | 170 (83%) | 18  (9%) | 86  (42%) | 16  (8%) | 84  (41%) |
| Child at T2^a^ | 77  (45%) | 93  (55%) | 43  (25%) | 43  (25%) | 34  (20%) | 50  (29%) |
| Pregnant at T2^a^ | 4  (2%) | 166 (98%) | 3  (2%) | 83  (49%) | 1  (1%) | 83  (49%) |
| Currently trying^a^ | 56  (33%) | 114 (67%) | 27  (16%) | 59  (35%) | 29  (17%) | 55  (32%) |
| Child or intention^a^ | 107  (63%) | 63  (37%) | 56  (33%) | 30  (18%) | 51  (30%) | 33  (19%) |

*Note.* ^a^ 34 participants who already had a child at T1 are not included anymore. At T2, participants were additionally asked if they are currently trying to have a/another child. Responses were made on a 7 point-Likert scale ranging from 1 (trying to avoid it) to 7 (trying to). All participants indicating an answer equal or larger than 4 are seen as currently trying to have a/another child.

## **B.** **Descriptive Data of Participants of the Sociosexuality Study**

**Participants and Procedure**

In addition to our main sample, we invited another 142 participants (♀ = 72, ♂ = 70; *M* = 24.1 years, *SD* = 2.9, range = 20 - 30) of a former study, the Sociosexuality Study (Penke & Asendorpf, 2008) (T1), to participate in our online study (referred to as T2). T1 took place from 2004 to 2006. We only invited single participants, who made up half of the sample of the Sociosexuality Study, owing to the fact that only their ideal partner preferences were assessed at T1. One of these initial participants also took part in the BSDS, hence we only included this person in the analyses reported in the main text, not in these supplementary analyses. At T2, we were able to re-recruit 66 individuals (dropout rate 54%). Because one person dropped out during our T2 online study, our final sample size consists out of 65 individuals (♀ = 31, ♂ = 34 male; *M* = 38.7 years, *SD* = 3.3, range = 34 - 45), with 28% being single at T2, 71% being in a relationship and 2% in an undefined relationship. At T2, 80% of participants had at least a university degree. At T1, none had children. Whereas at T2, 52% had at least one child or were currently pregnant, and 23% were currently trying to have a child. Attrition analyses did not reveal significant group differences between those participants who participated versus those who did not participate at T2 (Table S3).

**Table S3**

*Attrition analysis for the re-assessment of the Sociosexuality Study*

| Participation | Drop-outs | Re-recruited |  |
| --- | --- | --- | --- |
| *n* | 76 | 66 |  |
| Variable | *M* (*SD*) | *M* (*SD*) | *p* |
| Age | 23.92 (2.76) | 24.36 (3.12) | .37 |
| Sex. orientation | 1.20 (.40) | 1.23 (.46) | .68 |
| O | 3.85 (.68) | 3.90 (.54) | .65 |
| C | 3.46 (.61) | 3.38 (.63) | .45 |
| E | 3.56 (.79) | 3.61 (.64) | .68 |
| A | 3.31 (.56) | 3.37 (.57) | .52 |
| N | 2.81 (.73) | 2.81 (.76) | .97 |

*Note.* O = Openness; C = Conscientiousness; E = Extraversion; A = Agreeableness; N = Neuroticism; Being assessed using the BFI (Lang, Luedtke, & Asendorpf, 2001).

The procedure at T2 was exactly the same as reported for the BSDS in the main text. One important difference at T1 in the Sociosexuality Study is the assessment of ideal partner preferences. Former participants of the Sociosexuality study rated 13 items regarding how strongly they would like a certain characteristic to be pronounced in an ideal partner on a scale ranging from 1 = *very little* to 5 = *as strong as possible*. At T2, they now rated the importance of the same 13 items on a scale ranging from 1 = *very unimportant* to 5 = *very important*. Participants also rated 59 additional items, which were initially assessed in the other sample.

## **C.** **Preference Dimensions**

Because we could not foresee how many people would participate after around 14 years, we decided to recruit former participants of two different samples, despite the challenging fact that the measure of ideal partner preferences differed between the two samples. To overcome the difficulties of different measurements, we ran a pre-test. A new sample (*N* = 436, ♀ = 315 [72%], ♂ = 121 [28%]; *M* = 34.83 years, *SD* = 13.81 years) rated the initial items of both studies in its importance in an ideal partner and how strongly they would like a certain characteristic to be pronounced. We ran several principal component analyses (separately for each rating scale, a combination of both scales and separately for each sex) with oblimin rotation. An eight factor solution provided the best solution with its factors being: Status-resources, warmth-trustworthiness, vitality-attractiveness, intelligence, family orientation, humour, creativity and adventurousness-confidence. The idea of these preference dimensions was to give us the opportunity not to distinguish between the initial studies in our analysis. Instead we wanted to run our analysis for each preference dimension.

**Replication of Preference Dimensions**

In a confirmatory factor analysis (CFA), we investigated the fit of our assumed factor structure. Goodness of fit indexes revealed a mixed pattern: (χ² F(2441) = 6106.77, p > .001; standardized root mean square residuals [SRMR] = .099; root mean square errors of approximation [RMSEA] = .075, 90% CI[.072; .077]; comparative fit index [CFI] =.694; Tucker-Lewis Index [TLI] = .680). RMSEA and SRMR fell slightly below an acceptable range, TLI and CFI fell below the threshold of .90. Because fit indices only marginally increased in further exploration (i.e. excluding items with factor loadings smaller than .40) and with our initial factor solution being a compromise between different assessment methods making a better fit unlikely, we calculated participant’s mean preferences based on our initial factor structure (Table S4).

**Table S4**

*Exemplary items of each preference dimension. The first item is always an item used in the initial Sociosexuality Study with the rest of the items deriving from the former BSDS. One exception is the dimension humour which does not contain any item of the former Sociosexuality Study.*

| Dimension | Content |
| --- | --- |
| Status-resources | 1. occupational success/good occupational prospect 2. wealthy 3. financially secure 4. successful |
| Warmth- trustworthiness | 1. faithfulness/reliability 2. understanding 3. sensitive 4. trustworthy |
| Vitality-attractiveness | 1. physical attractiveness/sex-appeal 2. erotic 3. sexy 4. arousing |
| Family orientation | 1. parental abilities/whish for children 2. likes children 3. family-oriented 4. being a good mother/father |
| Intelligence | 1. intelligence 2. educated 3. sharp 4. clever |
| Humour | 1. fun 2. good sense of humour 3. shrewd 4. good fun |
| Creativity | 1. creativity 2. broad-minded 3. inventive 4. original |
| Adventurousness- Confidence | 1. adventurousness/activity 2. venturesome 3. assertive 4. confident |

## **D. Measurement Invariance**

Because at T2, we elaborated some more on our instruction to rate ideal partner preferences compared to the instruction at T1 (see Table S5), we tested for measurement invariance across the two time points. We oriented our check for measurement invariance on the procedure suggested by Mackinnon et al. (2022), hence we specified confirmatory factor analysis models with increasingly strict assumptions.

**Table S5**

*Instruction at T1 compared to T2*

| T1 | T2 |
| --- | --- |
| “On this page, you can find a list of characteristics which one may prefer in a partner. Please describe your ideal partner by rating each characteristic in how important you rate each in a partner from 1 to 5 on the following scale [...].” | “In the following, we are interested in your expectations of an ideal partner. In particular, we are interested in your expectations of an ideal partner for a committed long-term relationship. For this reason we will present you with a list of characteristics which a person may have. Please note: some of the characteristics will be presented in a similar way. This repetition is intended.  Now rate every characteristic in its importance for an ideal partner. Please use the following scale [...].”  Additional instruction which was presented if participants were currently in a relationship:  “Try to rate each characteristics independently of your current partner but rate each characteristic in your ideal expectation of a partner.” |

In a first model, we checked for configural invariance, testing whether the same factor structure of ideal partner preferences applies from T1 to T2. We evaluated the configural model based on the following parameters (Hu & Benteler, 1998):

- The Root Mean Square Error of Approximation (RMSEA) with a cutoff close to 0.06 and
- The Standardized Root Mean Squared Residual (SRMR) with a cutoff close to 0.08

With an RMSEA of 0.065 and a SRMR of 0.091, we assume that configural invariance is supported, meaning that free and fixed loadings largely follow the same pattern (Mackinnon et al., 2022). We then tested the model fit of a metric model compared to the configural model. Because model fit is significantly better, we assume that metric invariance is supported, meaning that item loadings are equivalent on each factor. Items do not become more representative for the construct over time. Third, we compared the fit of a scalar model to the metric model. However, the scalar model fit is worse compared to the metric model. Though, with an RMSEA of 0.066 and a SRMR of 0.102, this model only slightly exceeds our evaluation criteria (Table S6). Hence, we assume scalar invariance to be partially supported, suggesting that from T1 to T2, the interpretation of the absolute value of an item slightly but not dramatically changed. This means, for example, a response of 5 “extremely important”, slightly increased or decreased in its value over time. Because the assumption of scalar invariance is partly violated, checks for residual invariance (assuming that the specific variance and error variance are similar across both time points) are redundant.

**Table S6**

*Test for Measurement Invariance*

|  | Model | | | |
| --- | --- | --- | --- | --- |
|  | Configural | Metric | Scalar | Residual |
| No. of Estimated Parameters | 526 | 468 | 410 | 352 |
| Raw Loglikelihood | -25495 | -25545 | -25734 | -25775 |
| Δ χ2, p-value | N/A | 77.754, 0.0427 | 386.64, >.001 | 64.833, 0.250 |
| Robust CFI | 0.625 | 0.623 | 0.601 | 0.601 |
| Raw AIC | 52042 | 52026 | 52287 | 52254 |
| Δ AIC | N/A | -16 | +261 | -33 |
| Raw BIC | 53787 | 53579 | 53648 | 53422 |
| Δ BIC | N/A | -208 | +69 | -226 |
| Robust RMSEA | 0.065 | 0.065 | 0.066 | 0.066 |
| SRMR | 0.091 | 0.101 | 0.102 | 0.102 |

*Note.* Chi-square difference test (i.e., log likelihood ratio tests) values are calculated between the current column model and the preceding column model. AIC and BIC are calculated as the difference from the preceding model.

# **S2. Robustness Checks**

## **A.** **Rank-order Stability**

**Figure S3.**

*Plots of T1 preferences predicting T2 preferences, separately for each dimension*

*
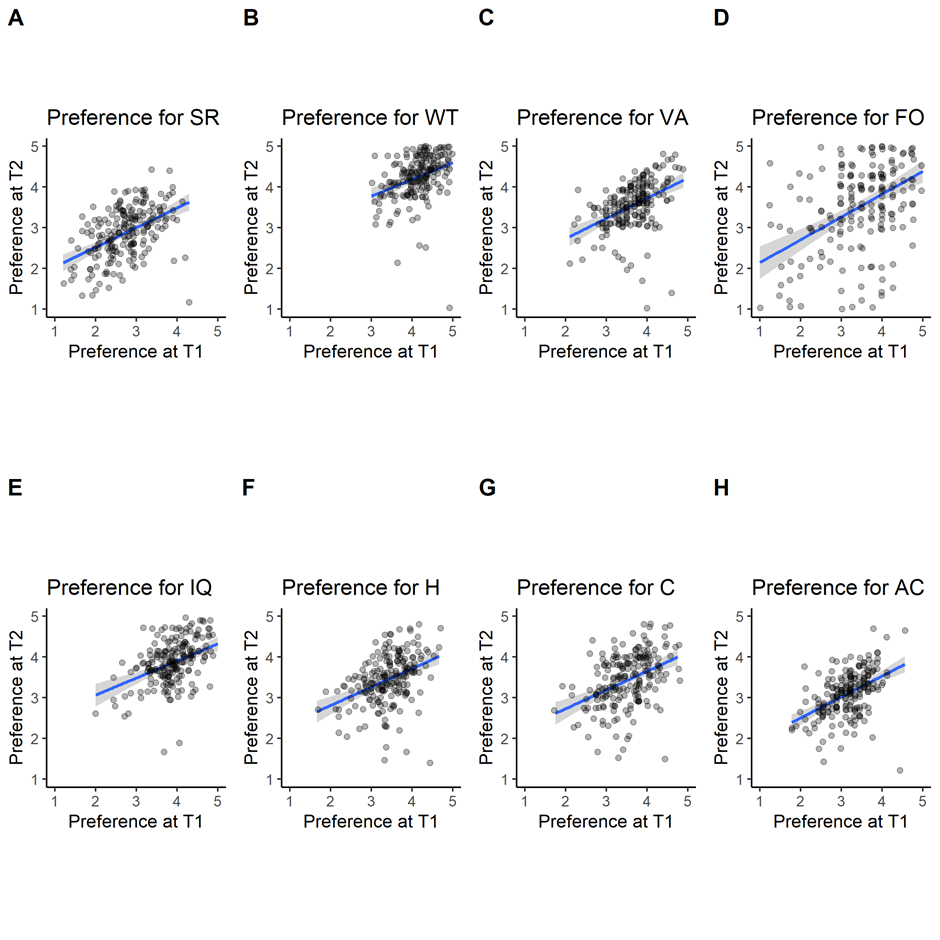
*

*Note.* The solid line in blue represents simple slopes derived from the regression models of former participants of the BSDS. The grey area around each line represents the 95% confidence interval. SR = status-resources, WT = warmth-trustworthiness, VA = vitality-attractiveness, FO = family orientation, IQ = intelligence, H = humour, C = creativity, AC = adventurousness-confidence.

**Table S7**

*Rank-order stability reported separately for each sex*

|  | male (*n* = 100) | | female (*n* = 104) | |
| --- | --- | --- | --- | --- |
|  | *r* [95% CI] | *p* | *r* [95% CI] | *p* |
| warmth-trustworthiness | .37 [.18; .52] | <.001 | .25 [.06; .42] | .012 |
| vitality-attractiveness | .44 [.26; .58] | <.001 | .33 [.15; .49] | .001 |
| status-resources | .55 [.39; .67] | <.001 | .33 [.14; .49] | .001 |
| family orientation | .48 [.31; .61] | <.001 | .44 [.28; .59] | <.001 |
| intelligence | .43 [.25; .58] | <.001 | .31 [.12; .47] | .002 |
| creativity | .35 [.17; .52] | <.001 | .43 [.26; .58] | <.001 |
| humour | .40 [.22; .55] | <.001 | .39 [.21; .54] | <.001 |
| adventurousness-confidence | .48 [.32; .62] | <.001 | .39 [.21; .54] | <.001 |
| Overall | .44 |  | .36 | .504 |

*Note.* *p* = *p*-values for each dimension indicate whether correlation coefficients are significant. The *p*-value in the column overall, indicated whether the mean rank-order stability differs between the sexes.

**Table S8**

*Means, standard Deviations, effect Sizes and correlations of T1 and T2 importance ratings separately for participants with and without children, not taking into account participants’ intention to have children.*

|  | with children (*n* =77) | | | | | without children (*n* = 93) | | | | |  |
| --- | --- | --- | --- | --- | --- | --- | --- | --- | --- | --- | --- |
|  | *M_T1 with_*  (*SD*_T1_ *_with_*) | *M_T2 with_*  (*SD*_T1_ *_with_*) | *p_with_* | *d_with_* | *r_with_* | *M_T1 without_* (*SD*_T1 without_) | *M_T2 without_* (*SD*_T2 without_) | *p_without_* | *d_without_* | *r_without_* | *p_comparison_* |
| warmth-trustworthiness | 4.21 (0.42) | 4.37 (0.59) | .042 | 0.24 | .16 | 4.10 (0.50) | 4.22 (0.52) | .022 | 0.24 | .55 | >.001 |
| vitality-attractiveness | 3.64 (0.55) | 3.55 (0.59) | .188 | 0.15 | .45 | 3.62 (0.53) | 3.58 (0.57) | .368 | 0.09 | .55 | .283 |
| status-resources | 2.71 (0.64) | 2.99 (0.59) | .001 | 0.41 | .39 | 2.69 (0.60) | 2.75 (0.63) | .422 | 0.08 | .50 | .263 |
| family orientation | 3.86 (0.79) | 4.31 (0.71) | >.001 | 0.46 | .14 | 3.19 (1.01) | 3.04 (1.21) | .229 | 0.13 | .45 | .016 |
| intelligence | 3.92 (0.53) | 3.93 (0.61) | .89 | 0.02 | .29 | 3.9 (0.54) | 3.86 (0.53) | .545 | 0.06 | .48 | .077 |
| creativity | 3.44 (0.55) | 3.42 (0.69) | .822 | 0.03 | .28 | 3.56 (0.59) | 3.46 (0.61) | .097 | 0.17 | .55 | .006 |
| humour | 3.56 (0.47) | 3.54 (0.56) | .844 | 0.02 | .16 | 3.34 (0.52) | 3.44 (0.63) | .111 | 0.17 | .53 | .001 |
| adventurousness-confidence | 3.16 (0.50) | 3.17 (0.52) | .86 | 0.02 | .37 | 3.15 (0.54) | 3.05 (0.60) | .097 | 0.17 | .55 | .048 |

*Note.* The lower case “with” refers to the group of participants with children, the lower case “without” refers to participants without children. *z*-transformed correlation coefficients of each group are compared using a z-test with the column *p_comparison_* referring to the *p*-values of each comparison.

## **B.** **Profile Stability**

As a robustness check, we calculated the profile stability between T1 and T2 preferences separately for each sex. We did so separately for each dimension (women: overall *r* = .70 (*t* (104) = 8.54, *p* <.001); distinctive *r* = .51 (*t* (104) = 8.99, *p* <.001); men: overall *r* = .76 (*t* (95) = 8.06, *p* <.001); distinctive *r* = .50 (*t* (95) = 8.56, *p* <.001) and on an item-level (women: overall *r* = .62 (*t* (101) = 11.33, *p* <.001); distinctive *r* = .35 (*t* (101) = 15.86, *p* <.001); men: overall *r* = .63 (*t* (95) = 10.86, *p* <.001); distinctive *r* = .35 (*t* (95) = 14.74, *p* <.001)

As suggested by a reviewer comment, we fitted a multilevel model in which we predicted T2 ideal partner ratings with T1 ideal partner ratings while including a random intercept for each item and participants’ IDs. T1 and T2 ratings were centred on their item means, separately for each timepoint. We found that T1 and T2 ratings were significantly correlated (*b* = 0.39, 95%CI [0.38, 0.41], *p* <.001), even after removing the normative rating of each item (Table S9).

**Table S9**

*Multilevel model predicting T2 ideals with participants’ T1 ideals.*

|  | **T2 Rating (centered)** | | | | |
| --- | --- | --- | --- | --- | --- |
| *Coeffcient* | *Estimates* | *std. Error* | *95% CI* | *Statistic* | *p-values* |
| Intercept | 0.00 | 0.03 | -0.05, 0.05 | 0.01 | .995 |
| T1 Rating (centered) | 0.39 | 0.01 | 0.38, 0.41 | 45.13 | **<.001** |
| **Random Effects** | | | | | |
| σ^2^ | 0.60 | | | | |
| τ_00_ _session_ | 0.14 | | | | |
| τ_00_ _item_ | 0.00 | | | | |
| ICC | 0.19 | | | | |
| N _session_ | 204 | | | | |
| N _item_ | 58 | | | | |
| Observations | 11824 | | | | |
| Marginal R^2^ / Conditional R^2^ | 0.138 / 0.298 | | | | |

##

## **C.** **Relationship of Sex with Mean-level Changes**

**Table S10**

*Multilevel models for the dimension warmth-trustworthiness, vitality-attractiveness, status-resources and family orientation*

|  | warmth-trustworthiness | | | vitality-attractiveness | | | status-resources | | | Family orientation | | |
| --- | --- | --- | --- | --- | --- | --- | --- | --- | --- | --- | --- | --- |
| Predictors | *b* | 95% CI | *p* | *b* | 95% CI | *p* | *b* | 95% CI | *p* | *b* | 95% CI | *p* |
| (Intercept) | 4.12 | 4.02 – 4.23 | <0.001 | 3.78 | 3.68 – 3.89 | <0.001 | 2.56 | 2.44 – 2.67 | <0.001 | 3.34 | 3.13 – 3.55 | <0.001 |
| timepoint | 0.05 | -0.07 – 0.17 | 0.413 | -0.05 | -0.16 – 0.07 | 0.423 | 0.17 | 0.04 – 0.29 | 0.009 | 0.16 | -0.05 – 0.38 | 0.138 |
| sex | 0.07 | -0.07 – 0.22 | 0.336 | -0.29 | -0.43 – -0.14 | <0.001 | 0.36 | 0.20 – 0.53 | <0.001 | 0.26 | -0.03 – 0.55 | 0.081 |
| time point*sex | 0.08 | -0.09 – 0.25 | 0.352 | -0.08 | -0.25 – 0.08 | 0.307 | -0.05 | -0.23 – 0.12 | 0.548 | -0.21 | -0.51 – 0.10 | 0.181 |
| Random Effects | | | | | | | | | | | | |
| σ^2^ | 0.20 | | | 0.18 | | | 0.20 | | | 0.61 | | |
| τ_00_ | 0.08 _session_ | | | 0.11 _session_ | | | 0.15 _session_ | | | 0.50 _session_ | | |
| ICC | 0.30 | | | 0.39 | | | 0.43 | | | 0.45 | | |
| N | 204 _session_ | | | 204 _session_ | | | 204 _session_ | | | 204 _session_ | | |
| Observations | 408 | | | 408 | | | 408 | | | 408 | | |
| Marginal R^2^ / Conditional R^2^ | 0.020 / 0.312 | | | 0.093 / 0.446 | | | 0.086 / 0.480 | | | 0.008 / 0.455 | | |

*Note*. *b* = Estimate, 95% CI = 95% confidence interval, *p* = *p*-value. Preferences are predicted with the assessment time point (0 = T1, 1 = T2), sex (0 = male, 1 = female) and their interaction. A random effect is specified for each person.

**Table S11**

*Multilevel models for the dimension intelligence, creativity, adventurousness-confidence and humour*

|  | intelligence | | | creativity | | | adventurousness-confidence | | | humour | | |
| --- | --- | --- | --- | --- | --- | --- | --- | --- | --- | --- | --- | --- |
| Predictors | *b* | 95% CI | *p* | *b* | 95% CI | *p* | b | 95% CI | *p* | *b* | 95% CI | *p* |
| (Intercept) | 3.80 | 3.70 – 3.91 | <0.001 | 3.53 | 3.41 – 3.65 | <0.001 | 3.00 | 2.90 – 3.10 | <0.001 | 3.37 | 3.25 – 3.48 | <0.001 |
| time point | 0.01 | -0.11 – 0.13 | 0.829 | -0.06 | -0.20 – 0.07 | 0.344 | -0.01 | -0.11 – 0.10 | 0.919 | 0.04 | -0.08 – 0.17 | 0.493 |
| sex | 0.16 | 0.01 – 0.31 | 0.040 | -0.03 | -0.20 – 0.14 | 0.751 | 0.32 | 0.18 – 0.46 | <0.001 | 0.08 | -0.07 – 0.24 | 0.297 |
| time point*sex | -0.08 | -0.25 – 0.09 | 0.351 | -0.04 | -0.23 – 0.15 | 0.654 | -0.10 | -0.25 – 0.05 | 0.193 | -0.01 | -0.18 – 0.17 | 0.942 |
| Random Effects | | | | | | | | | | | | |
| σ^2^ | 0.19 | | | 0.24 | | | 0.15 | | | 0.20 | | |
| τ_00_ | 0.11 _session_ | | | 0.15 _session_ | | | 0.11 _session_ | | | 0.13 _session_ | | |
| ICC | 0.37 | | | 0.39 | | | 0.43 | | | 0.39 | | |
| N | 204 _session_ | | | 204 _session_ | | | 204 _session_ | | | 204 _session_ | | |
| Observations | 408 | | | 408 | | | 408 | | | 408 | | |
| Marginal R^2^ / Conditional R^2^ | 0.013 / 0.376 | | | 0.007 / 0.394 | | | 0.071 / 0.472 | | | 0.006 / 0.394 | | |

*Note. b* = Estimate, 95% CI = 95% confidence interval, *p* = *p*-value. Preferences are predicted with the assessment time point (0 = T1, 1 = T2), sex (0 = male, 1 = female) and their interaction. A random effect is specified for each person.

## **D.** **Relationship of Age with Mean-level Changes**

**Table S12**

*Multilevel models for the dimension warmth-trustworthiness, vitality-attractiveness, status-resources and family orientation*

|  | warmth-trustworthiness | | | vitality-attractiveness | | | status-resources | | | family orientation | | |
| --- | --- | --- | --- | --- | --- | --- | --- | --- | --- | --- | --- | --- |
| Predictors | *b* | 95% CI | *p* | *b* | 95% CI | *p* | b | 95% CI | *p* | *b* | 95% CI | *p* |
| (Intercept) | 4.16 | 4.09 – 4.23 | <0.001 | 3.64 | 3.56 – 3.71 | <0.001 | 2.74 | 2.66 – 2.83 | <0.001 | 3.47 | 3.34 – 3.60 | <0.001 |
| time point | 0.09 | 0.01 – 0.18 | 0.034 | -0.09 | -0.17 – -0.01 | 0.027 | 0.14 | 0.05 – 0.22 | 0.002 | 0.06 | -0.09 – 0.21 | 0.442 |
| age at T1 | -0.02 | -0.10 – 0.05 | 0.527 | 0.06 | -0.02 – 0.14 | 0.135 | 0.08 | -0.01 – 0.16 | 0.078 | -0.27 | -0.41 – -0.14 | <0.001 |
| time point*age | -0.05 | -0.14 – 0.03 | 0.243 | -0.11 | -0.19 – -0.03 | 0.007 | -0.10 | -0.18 – -0.01 | 0.029 | -0.24 | -0.39 – -0.09 | 0.002 |
| Random Effects | | | | | | | | | | | | |
| σ^2^ | 0.19 | | | 0.17 | | | 0.20 | | | 0.59 | | |
| τ_00_ | 0.08 _session_ | | | 0.14 _session_ | | | 0.18 _session_ | | | 0.36 _session_ | | |
| ICC | 0.30 | | | 0.45 | | | 0.48 | | | 0.38 | | |
| N | 204 _session_ | | | 204 _session_ | | | 204 _session_ | | | 204 _session_ | | |
| Observations | 408 | | | 408 | | | 408 | | | 408 | | |
| Marginal R^2^ / Conditional R^2^ | 0.018 / 0.313 | | | 0.016 / 0.462 | | | 0.020 / 0.491 | | | 0.150 / 0.475 | | |

*Note.* *b* = Estimate, 95% CI = 95% confidence interval, *p* = *p*-value. Preferences are predicted with the assessment time point (0 = T1, 1 = T2), age (z-standardised) and their interaction. A random effect is specified for each person.

**Table S13**

*Multilevel models for the dimension intelligence, creativity, humour and adventurousness-confidence*

|  | intelligence | | | creativity | | | humour | | | adventurousness-confidence | | |
| --- | --- | --- | --- | --- | --- | --- | --- | --- | --- | --- | --- | --- |
| Predictors | *b* | 95% CI | *p* | *b* | 95% CI | *p* | *b* | 95% CI | *p* | *b* | 95% CI | *p* |
| (Intercept) | 3.89 | 3.81 – 3.96 | <0.001 | 3.52 | 3.43 – 3.60 | <0.001 | 3.41 | 3.33 – 3.49 | <0.001 | 3.16 | 3.09 – 3.23 | <0.001 |
| time point | -0.03 | -0.11 – 0.06 | 0.520 | -0.09 | -0.18 – 0.01 | 0.070 | 0.04 | -0.05 – 0.13 | 0.365 | -0.06 | -0.13 – 0.02 | 0.140 |
| age at T1 | -0.09 | -0.17 – -0.02 | 0.016 | 0.03 | -0.06 – 0.11 | 0.542 | -0.08 | -0.16 – -0.00 | 0.040 | -0.02 | -0.09 – 0.06 | 0.645 |
| time point*age | -0.03 | -0.12 – 0.05 | 0.436 | -0.02 | -0.12 – 0.07 | 0.604 | -0.04 | -0.13 – 0.05 | 0.356 | -0.06 | -0.13 – 0.01 | 0.113 |
| Random Effects | | | | | | | | | | | | |
| σ^2^ | 0.19 | | | 0.24 | | | 0.20 | | | 0.15 | | |
| τ_00_ | 0.10 _session_ | | | 0.15 _session_ | | | 0.12 _session_ | | | 0.13 _session_ | | |
| ICC | 0.35 | | | 0.39 | | | 0.38 | | | 0.47 | | |
| N | 204 _session_ | | | 204 _session_ | | | 204 _session_ | | | 204 _session_ | | |
| Observations | 408 | | | 408 | | | 408 | | | 408 | | |
| Marginal R^2^ / Conditional R^2^ | 0.040 / 0.375 | | | 0.006 / 0.394 | | | 0.034 / 0.396 | | | 0.014 / 0.475 | | |

*Note.* *b* = Estimate, 95% CI = 95% confidence interval, *p* = *p*-value. Preferences are predicted with the assessment time point (0 = T1, 1 = T2), age (*z*-standardised) and their interaction. A random effect is specified for each person.

## **E.** **Relationship of Parenthood and Mean-level Changes**

**Table S14**

*Multilevel models for the dimension warmth-trustworthiness, vitality-attractiveness, status-resources and family orientation*

|  | warmth-trustworthiness | | | vitality-attractiveness | | | status-resources | | | family orientation | | |
| --- | --- | --- | --- | --- | --- | --- | --- | --- | --- | --- | --- | --- |
| Predictors | *b* | 95% CI | *p* | *b* | 95% CI | *p* | *b* | 95% CI | *p* | *b* | 95% CI | *p* |
| (Intercept) | 4.08 | 3.96 – 4.21 | <0.001 | 3.61 | 3.48 – 3.75 | <0.001 | 2.74 | 2.59 – 2.89 | <0.001 | 2.89 | 2.67 – 3.10 | <0.001 |
| time point | 0.10 | -0.04 – 0.25 | 0.161 | -0.02 | -0.16 – 0.11 | 0.739 | 0.09 | -0.07 – 0.25 | 0.263 | -0.31 | -0.58 – -0.05 | 0.022 |
| Child at T2 | 0.10 | -0.06 – 0.26 | 0.201 | 0.03 | -0.15 – 0.20 | 0.768 | -0.06 | -0.25 – 0.13 | 0.537 | 0.96 | 0.69 – 1.23 | <0.001 |
| time point * Child at T2 | 0.05 | -0.13 – 0.23 | 0.553 | -0.07 | -0.25 – 0.10 | 0.425 | 0.10 | -0.11 – 0.30 | 0.350 | 0.70 | 0.36 – 1.03 | <0.001 |
| Random Effects | | | | | | | | | | | | |
| σ^2^ | 0.17 | | | 0.16 | | | 0.21 | | | 0.59 | | |
| τ_00_ | 0.10 _session_ | | | 0.16 _session_ | | | 0.17 _session_ | | | 0.17 _session_ | | |
| ICC | 0.36 | | | 0.50 | | | 0.45 | | | 0.23 | | |
| N | 170 _session_ | | | 170 _session_ | | | 170 _session_ | | | 170 _session_ | | |
| Observations | 340 | | | 340 | | | 340 | | | 340 | | |
| Marginal R^2^ / Conditional R^2^ | 0.033 / 0.386 | | | 0.005 / 0.504 | | | 0.016 / 0.458 | | | 0.362 / 0.507 | | |

*Note.* *b* = Estimate, 95% CI = 95% confidence interval, *p* = *p*-value. Preferences are predicted with the assessment time point (0 = T1, 1 = T2) and whether participants have children at T2 (0 = no children, 1 = children) and their interaction. A random effect is specified for each person.

**Table S15**

*Multilevel models for the dimension intelligence, creativity, adventurousness confidence and humour*

|  | intelligence | | | creativity | | | adventurousness confidence | | | humour | | |
| --- | --- | --- | --- | --- | --- | --- | --- | --- | --- | --- | --- | --- |
| Predictors | *b* | 95% CI | *p* | *b* | 95% CI | *p* | *b* | 95% CI | *p* | *b* | 95% CI | *p* |
| (Intercept) | 3.85 | 3.72 – 3.99 | <0.001 | 3.50 | 3.35 – 3.65 | <0.001 | 3.12 | 2.98 – 3.25 | <0.001 | 3.28 | 3.14 – 3.42 | <0.001 |
| time point | 0.03 | -0.12 – 0.18 | 0.730 | -0.02 | -0.19 – 0.14 | 0.775 | -0.04 | -0.18 – 0.10 | 0.564 | 0.13 | -0.03 – 0.28 | 0.103 |
| Child at T2 | 0.09 | -0.08 – 0.26 | 0.308 | 0.00 | -0.19 – 0.20 | 0.962 | 0.06 | -0.11 – 0.23 | 0.471 | 0.25 | 0.08 – 0.43 | 0.004 |
| time point * child at T2 | -0.06 | -0.25 – 0.13 | 0.509 | -0.06 | -0.27 – 0.14 | 0.552 | -0.01 | -0.18 – 0.16 | 0.917 | -0.13 | -0.32 – 0.06 | 0.185 |
| Random Effects | | | | | | | | | | | | |
| σ^2^ | 0.18 | | | 0.22 | | | 0.16 | | | 0.19 | | |
| τ_00_ | 0.12 _session_ | | | 0.16 _session_ | | | 0.14 _session_ | | | 0.11 _session_ | | |
| ICC | 0.39 | | | 0.42 | | | 0.47 | | | 0.37 | | |
| N | 170 _session_ | | | 170 _session_ | | | 170 _session_ | | | 170 _session_ | | |
| Observations | 340 | | | 340 | | | 340 | | | 340 | | |
| Marginal R^2^ / Conditional R^2^ | 0.003 / 0.390 | | | 0.004 / 0.424 | | | 0.004 / 0.474 | | | 0.031 / 0.392 | | |

*Note. b* = Estimate, 95% CI = 95% confidence interval, *p* = *p*-value. Preferences are predicted with the assessment time point (0 = T1, 1 = T2) and whether participants have children at T2 (0 = no children, 1 = children) and their interaction. A random effect is specified for each person.

## **F.** **Insight into Preference Change**

**Figure S4**

*Histogram of participants’ perception of change*

*
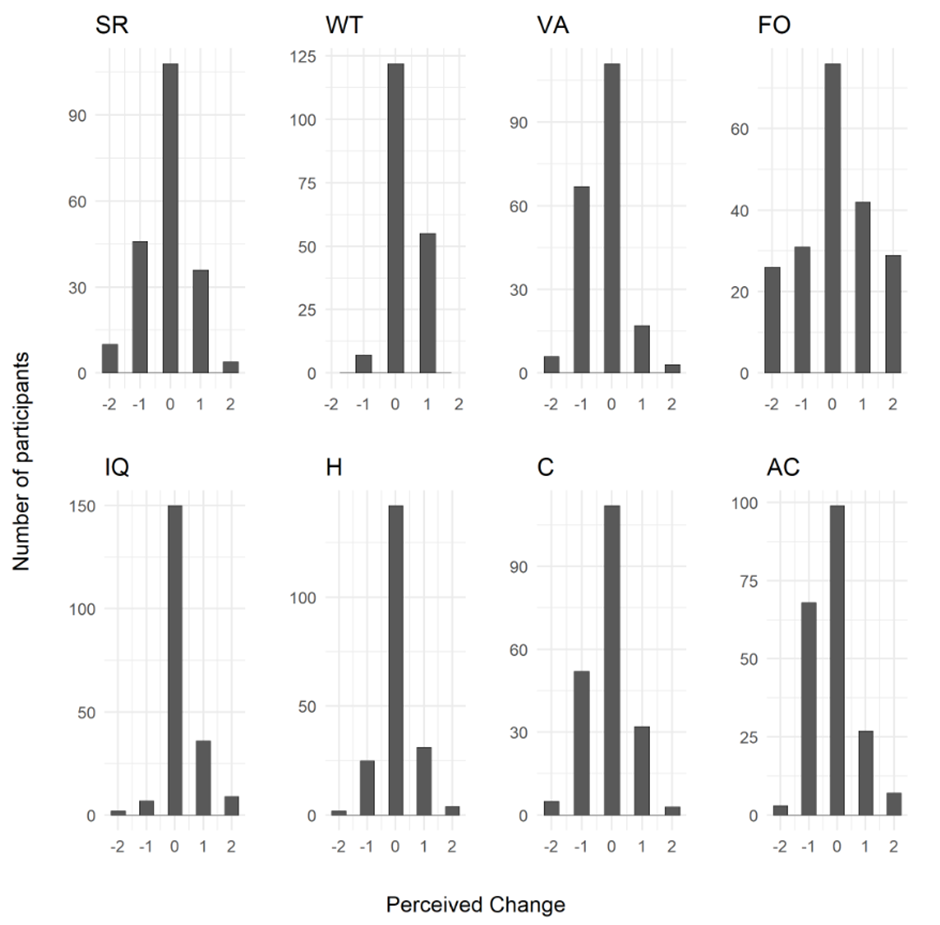
*

*Note*. Negative values indicate a reduced importance, zero indicates no change and positive values indicate an increased importance of the corresponding preference dimensions. SR = status-resources, WT = warmth-trustworthiness, VA = vitality-attractiveness, FO = family orientation, IQ = intelligence, H = humour, C = creativity, AC = adventurousness-confidence.

## **G. Relationship with Age**

**Table S16**

*Linear models investigating whether age is associated with participants’ insight into preference changes for the dimension warmth-trustworthiness, vitality-attractiveness, status-resources and family orientation.*

|  | warmth-trustworthiness | | | vitality-attractiveness | | | status-resources | | | family orientation | | |
| --- | --- | --- | --- | --- | --- | --- | --- | --- | --- | --- | --- | --- |
| Predictors | *b* | 95% CI | *p* | *b* | 95% CI | *p* | *b* | 95% CI | *p* | *b* | 95% CI | *p* |
| (Intercept) | 0.09 | 0.01 – 0.18 | 0.032 | -0.09 | -0.17 – -0.01 | 0.026 | 0.14 | 0.05 – 0.23 | 0.003 | 0.01 | -0.14 – 0.16 | 0.893 |
| perceived change | 0.12 | 0.03 – 0.21 | 0.008 | 0.12 | 0.04 – 0.20 | 0.005 | 0.04 | -0.05 – 0.13 | 0.409 | 0.50 | 0.35 – 0.65 | <0.001 |
| age | -0.06 | -0.14 – 0.03 | 0.174 | -0.11 | -0.19 – -0.03 | 0.006 | -0.08 | -0.17 – 0.01 | 0.095 | -0.02 | -0.17 – 0.14 | 0.818 |
| perceived change * age | 0.01 | -0.08 – 0.09 | 0.884 | 0.01 | -0.08 – 0.09 | 0.884 | 0.00 | -0.08 – 0.09 | 0.932 | -0.11 | -0.26 – 0.05 | 0.177 |
| Observations | 204 | | | 204 | | | 204 | | | 204 | | |
| R^2^ / R^2^ adjusted | 0.048 / 0.033 | | | 0.079 / 0.065 | | | 0.024 / 0.009 | | | 0.213 / 0.201 | | |

*Note.* *b* = Estimate, 95% CI = 95% confidence interval, *p* = *p*-value. Perceived change and age were *z*-standardised.

**Table S17**

*Linear models investigating whether age is associated with participants’ insight into preference changes for the dimension intelligence, creativity, humour and adventurousness-confidence.*

|  | intelligence | | | creativity | | | humour | | | adventurousness-confidence | | |
| --- | --- | --- | --- | --- | --- | --- | --- | --- | --- | --- | --- | --- |
| Predictors | *b* | 95% CI | *p* | *b* | 95% CI | *p* | *b* | 95% CI | *p* | *b* | 95% CI | *p* |
| (Intercept) | -0.04 | -0.13 – 0.05 | 0.364 | -0.09 | -0.18 – 0.01 | 0.070 | 0.04 | -0.05 – 0.13 | 0.343 | -0.06 | -0.13 – 0.02 | 0.127 |
| perceived change | 0.05 | -0.04 – 0.13 | 0.292 | 0.17 | 0.08 – 0.27 | <0.001 | 0.09 | 0.00 – 0.18 | 0.049 | 0.09 | 0.02 – 0.17 | 0.018 |
| age | -0.04 | -0.13 – 0.05 | 0.352 | -0.07 | -0.16 – 0.02 | 0.139 | -0.04 | -0.13 – 0.04 | 0.319 | -0.06 | -0.14 – 0.01 | 0.103 |
| perceived change * age | -0.06 | -0.14 – 0.02 | 0.112 | -0.01 | -0.09 – 0.07 | 0.743 | 0.02 | -0.08 – 0.11 | 0.708 | -0.03 | -0.11 – 0.05 | 0.402 |
| Observations | 204 | | | 204 | | | 204 | | | 204 | | |
| R^2^ / R^2^ adjusted | 0.024 / 0.009 | | | 0.067 / 0.053 | | | 0.027 / 0.012 | | | 0.049 / 0.035 | | |

*Note.* *b* = Estimate, 95% CI = 95% confidence interval, *p* = *p*-value. Perceived change and age were *z*-standardised.

## **H. Relationship with Sex**

**Table S18**

*Linear models investigating whether sex effects participant’s insight into preference changes for the dimension warmth-trustworthiness, vitality-attractiveness, status-resources and family-orientation.*

|  | warmth-trustworthiness | | | vitality-attractiveness | | | status-resources | | | family orientation | | |
| --- | --- | --- | --- | --- | --- | --- | --- | --- | --- | --- | --- | --- |
| Predictors | *b* | 95% CI | *p* | *b* | 95% CI | *p* | *b* | 95% CI | *p* | *b* | 95% CI | *p* |
| (Intercept) | 0.05 | -0.07 – 0.17 | 0.440 | -0.06 | -0.17 – 0.06 | 0.338 | 0.16 | 0.04 – 0.29 | 0.010 | 0.17 | -0.02 – 0.36 | 0.082 |
| perceived change | 0.13 | 0.01 – 0.26 | 0.032 | 0.08 | -0.02 – 0.19 | 0.129 | -0.02 | -0.16 – 0.13 | 0.837 | 0.65 | 0.44 – 0.85 | <0.001 |
| sex | 0.09 | -0.08 – 0.26 | 0.310 | -0.06 | -0.22 – 0.10 | 0.469 | -0.06 | -0.24 – 0.11 | 0.492 | -0.22 | -0.49 – 0.05 | 0.113 |
| perceived change * sex | -0.02 | -0.19 – 0.15 | 0.826 | 0.08 | -0.08 – 0.24 | 0.324 | 0.12 | -0.06 – 0.31 | 0.188 | -0.26 | -0.53 – 0.01 | 0.061 |
| Observations | 204 | | | 204 | | | 204 | | | 204 | | |
| R^2^ / R^2^ adjusted | 0.044 / 0.030 | | | 0.050 / 0.036 | | | 0.020 / 0.005 | | | 0.229 / 0.217 | | |

*Note.* *b* = Estimate, 95% CI = 95% confidence interval, *p* = *p*-value. Perceived change was *z*-standardised. Sex was dummy-coded (0 = male, 1= female).

**Table S19**

*Linear models investigating whether sex effects participant’s insight into preference changes for the dimension intelligence, creativity, humour and adventurousness-confidence.*

|  | intelligence | | | creativity | | | humour | | | adventurousness-confidence | | |
| --- | --- | --- | --- | --- | --- | --- | --- | --- | --- | --- | --- | --- |
| Predictors | *b* | 95% CI | *p* | *b* | 95% CI | *p* | *b* | 95% CI | *p* | *b* | 95% CI | *p* |
| (Intercept) | 0.03 | -0.09 – 0.15 | 0.667 | -0.09 | -0.23 – 0.04 | 0.170 | 0.05 | -0.07 – 0.18 | 0.400 | -0.01 | -0.11 – 0.10 | 0.900 |
| perceived change | 0.13 | 0.01 – 0.26 | 0.033 | 0.20 | 0.06 – 0.33 | 0.005 | 0.13 | 0.00 – 0.25 | 0.045 | 0.08 | -0.02 – 0.19 | 0.130 |
| sex | -0.09 | -0.26 – 0.08 | 0.283 | 0.00 | -0.18 – 0.19 | 0.968 | -0.02 | -0.19 – 0.15 | 0.815 | -0.10 | -0.24 – 0.05 | 0.202 |
| perceived change * sex | -0.14 | -0.31 – 0.03 | 0.098 | -0.06 | -0.25 – 0.13 | 0.512 | -0.07 | -0.24 – 0.11 | 0.450 | 0.03 | -0.12 – 0.18 | 0.719 |
| Observations | 204 | | | 204 | | | 204 | | | 204 | | |
| R^2^ / R^2^ adjusted | 0.027 / 0.012 | | | 0.058 / 0.044 | | | 0.024 / 0.009 | | | 0.041 / 0.027 | | |

*Note.* *b* = Estimate, 95% CI = 95% confidence interval, *p* = *p*-value. Perceived change was *z*-standardised. Sex was dummy-coded (0 = male, 1= female).

## **I. Relationship with Relationship Status**

Since T1, participants entered on average *M* = 1.6 (*SD* = 1.3, range = 0 – 7) romantic relationships lasting longer than 6 months. The mean duration of these relationships was 60.4 months (*SD* = 50.3, range = 6 – 160). 25 participants (12%) did not enter any romantic relationship lasting longer than 6 months, 94 participants (46%) entered one romantic relationship, 47 participants (23%) entered two romantic relationships, 21 participants (10%) entered three romantic relationships, 11 participants (5%) entered four romantic relationships, one participant (0.5%) entered five romantic relationships, two participants (1%) entered six romantic relationships and three participants (1%) entered seven romantic relationships lasting longer than 6 months since T1.

Because studies have shown that individuals adjust their ideal partner preferences according to their partner (e.g. Gerlach et al., 2019; Fletcher et al., 2000), we investigated the stability and change in regard to how many relationships participants had entered in the time between T1 and T2. Because subgroups are too small to be analysed separately, we divided participants into three groups: 25 participants (12.3%) who did not enter any relationship lasting longer than six months since T1 (referred to as no relationship), 94 participants (46.1%) who entered one relationship lasting longer than 6 months since T1 (referred to as one relationship), and 85 participants (41.7%) who entered more than one relationship lasting longer than 6 months since T1 (referred to as more relationships).

**Rank-order Stability**

We found no significant difference (*p* = .089) between the overall rank-order stability of participants who entered one relationship lasting longer than 6 months over the investigated 13 years (*r* = .34) and participants who entered more than one relationship lasting longer than 6 months (*r* = .50) (Table S20). Since the group of participants who did not enter any relationship lasting longer than 6 months is fairly small (*n* = 25), we do not interpret these correlation coefficients with the two other groups.

**Table S20**

*Correlations of T1 and T2 Preferences Reported Separately Regarding Participants’ Relationship Status*

|  | No relationship  *r* [95% CI] | One relationship  *r* [95% CI] | More relationships  *r* [95% CI] | | |
| --- | --- | --- | --- | --- | --- |
| warmth-trustworthiness | .55 [.19, .77] | .23 [.03, .41] | | .40 [.20, .56] |  |
| vitality-attractiveness | .44 [.05, .71] | .28 [.08, .45] | | .63 [.49, .75] |  |
| status-resources | .66 [.36, .84] | .44 [.26, .59] | | .44 [.26, .60] |  |
| family orientation | .03 [-.37, .42] | .41 [.22, .56] | | .60 [.45, .72] |  |
| intelligence | .45 [.06, .72] | .30 [.11, .48] | | .48 [.29, .63] |  |
| creativity | .53 [.18, .77] | .23 [.03, .41] | | .51 [.33, .65] |  |
| humour | .30 [-.11, .62] | .30 [.10, .47] | | .52 [.34, .66] |  |
| adventurousness-confidence | .56 [.21, .78] | .50 [.33, .64] | | .42 [.23, .58] |  |
| Overall | .46 | .34 | | .50 |  |

*Note.* No relationship *n* = 25, one relationship *n* = 94, more relationships *n* = 85.

**Profile Correlations**

The overall as well as the distinctive stability of participants’ profile correlations is comparable over all three groups (Table S21).

**Table S21**

*Profile Correlations of T1 and T2 Preferences Reported Separately Regarding Participants’ Relationship Status*

|  | No relationship | | One relationship | | More relationships | |
| --- | --- | --- | --- | --- | --- | --- |
|  | *r* | *p* | *r* | *p* | *r* | *p* |
| Item level | | | | | | |
| Overall correlation | .65 | <.001 | .63 | <.001 | .64 | <.001 |
| Distinctive stability | .40 | <.001 | .38 | <.001 | .42 | <.001 |
| Dimension level | | | | | | |
| Overall correlation | .90 | <.001 | .84 | <.001 | .75 | <.001 |
| Distinctive stability | .50 | <.001 | .50 | <.001 | .58 | <.001 |

*Note.* No relationship *n* = 25, one relationship *n* = 94, more relationships *n* = 85.

**Mean-level Changes**

Due to the small number of participants who did not enter any relationship lasting longer than 6 months, we do not interpret these results, but only compare results between the group of participants who entered one relationship and the group of participants who entered more than one relationship lasting longer than 6 months. Identical to our main results, we found the increased preference for warmth-trustworthiness and status-resources in the group of participants with more than one relationship. Results of the group of participants who entered one relationship slightly differed: Whereas we also found an increased preference for status-resources over time, participants also reported a significantly increased preference for family orientation over time but not for warmth-trustworthiness (see Table S22).

**Table S22**

*Means, Standard Deviations and Effect Sizes with Confidence Intervals of T1 and T2 Importance Ratings Reported Separately Regarding Participants’ Relationship Status*

|  | *No relationship* | | | | *One Relationship* | | | | *More Relationships* | | | |
| --- | --- | --- | --- | --- | --- | --- | --- | --- | --- | --- | --- | --- |
|  | *M*_T1_ (*SD*_T1_) | *M*_T2_ (*SD*_T2_) | *p* | *d*  *[95% CI]* | *M*_T1_ (*SD*_T1_) | *M*_T1_ (*SD*_T1_) | *p* | *d*  *[95% CI]* | *M*_T1_ (*SD*_T1_) | *M*_T2_ (*SD*_T2_) | *p* | *d*  *[95% CI]* |
| warmth-trustworthiness | 4.23 (0.46) | 4.20 (0.61) | .770 | -0.06  [-0.44, 0.33] | 4.18 (0.43) | 4.22 (0.68) | .633 | 0.06  [-0.19, 0.31] | 4.12 (0.48) | 4.31 (0.48) | .001 | 0.40  [0.16, 0.65] |
| vitality-attractiveness | 3.51 (0.61) | 3.41 (0.62) | .470 | -0.16  [-0.59, 0.27] | 3.67 (0.51) | 3.51 (0.59) | .025 | -0.28  [-0.53, -0.03] | 3.64 (0.52) | 3.62 (0.58) | .759 | -0.03  [-0.21, 0.15] |
| status-resources | 2.79 (0.67) | 2.77 (0.72) | .862 | -0.03  [-0.36, 0.30] | 2.73 (0.61) | 2.86 (0.61) | .050 | 0.22  [0.00, 0.44] | 2.74 (0.60) | 2.94 (0.61) | .007 | 0.32  [0.09, 0.55] |
| family orientation | 3.56 (0.85) | 3.32 (1.06) | .380 | -0.25  [-0.82, 0.32] | 3.46 (0.91) | 3.70 (1.05) | .032 | 0.24  [0.02, 0.47] | 3.46 (1.02) | 3.40 (1.28) | .625 | -0.05  [-0.24, 0.14] |
| intelligence | 3.91 (0.53) | 3.89 (0.59) | .911 | -0.02  [-0.45, 0.40] | 3.87 (0.56) | 3.85 (0.66) | .723 | -0.04  [-0.28, 0.20] | 3.89 (0.48) | 3.86 (0.48) | .532 | -0.07  [-0.29, 0.15] |
| creativity | 3.59 (0.62) | 3.46 (0.66) | .306 | -0.20  [-0.59, 0.19] | 3.56 (0.52) | 3.45 (0.67) | .141 | -0.19  [-0.44, 0.06] | 3.45 (0.61) | 3.41 (0.67) | .525 | -0.07  [-0.28, 0.14] |
| humour | 3.29 (0.52) | 3.27 (0.68) | .891 | -0.03  [-0.51, 0.44] | 3.46 (0.52) | 3.47 (0.58) | .876 | 0.02  [-0.22, 0.26] | 3.39 (0.55) | 3.48 (0.62) | .151 | 0.15  [-0.06, 0.37] |
| adventurousness-confidence | 3.19 (0.55) | 2.99 (0.59) | .081 | -0.34  [-0.73, 0.05] | 3.14 (0.49) | 3.13 (0.53) | .807 | 0.03  [-0.23, 0.18] | 3.17 (0.50) | 3.11 (0.57) | .319 | -0.12  [-0.35, 0.11] |

*Note.* *p* = *p*-values of two-sided t-tests in which we compared participant’s mean preferences at T1 and T2.

## **J. Relationship with Relationship Length**

For each participant, we extracted participants’ individual distinctive profile correlation. Using a multilevel model, we predicted each relationship length with participants’ distinctive profile correlation. Because participants could have entered more than one relationship, we included a random intercept for participants IDs. We repeated these analyses in a second model in which we used participants’ overall profile correlation instead of their distinctive profile correlation. We could not find a significant association between participants’ distinctive profile correlation or overall profile correlation and their relationship length (see Table S23 and Table S24).

**Table S23**

*Multilevel Model Predicting Relationship Length with Participants’ Distinctive Profile Correlation*

|  | Relationship Length (Months) | | | | |
| --- | --- | --- | --- | --- | --- |
| *Coeffcient* | *Estimates* | *std. Error* | *95% CI* | *Statistic* | *p-values* |
| Intercept | 77.10 | 7.79 | 61.82, 92.37 | 9.89 | **<.001** |
| Distinctive correlation | -21.81 | 17.71 | -56.52, 12.89 | -1.23 | .218 |
| **Random Effects** | | | | | |
| σ^2^ | 1647.73 | | | | |
| τ_00_ _session_ | 1038.38 | | | | |
| ICC | 0.39 | | | | |
| N _session_ | 172 | | | | |
| Observations | 319 | | | | |
| Marginal R^2^ / Conditional R^2^ | 0.006 / 0.391 | | | | |

**Table S24**

*Multilevel Model Predicting Relationship Length with Participants‘ Overall Profile Correlation*

|  | Relationship Length (Months) | | | | |
| --- | --- | --- | --- | --- | --- |
| *Coeffcient* | *Estimates* | *std. Error* | *95% CI* | *Statistic* | *p-values* |
| Intercept | 62.36 | 12.80 | 37.29, 87.44 | 4.87 | **<.001** |
| Overall correlation | 10.00 | 20.09 | -29.36, 49.37 | 0.50 | .618 |
| **Random Effects** | | | | | |
| σ^2^ | 1655.12 | | | | |
| τ_00_ _session_ | 1041.16 | | | | |
| ICC | 0.39 | | | | |
| N _session_ | 172 | | | | |
| Observations | 319 | | | | |
| Marginal R^2^ / Conditional R^2^ | 0.001 / 0.387 | | | | |

# **S3. Results for each study and across studies**

## **A.** **Rank-Order Stability**

In Table S25, we calculated the correlation of T1 and T2 preferences once for both samples, only for the former participants of the Sociosexuality Study. However, results of former participants of the Sociosexuality Study should be interpreted with caution due to the limited number of items assessing T1 preferences (13 items) and the small sample size (*n* = 65).

When comparing our main analyses to analyses on former participants of the Sociosexuality Study, descriptively, we found an overall lower rank-order stability as well as lower rank-order stabilities for each dimension (see Table S25)

**Table S25**

*Correlation coefficients between T1 and T2 preferences for the whole sample and separately for each initial study.*

|  | Both samples | | Sociosexuality study | | Main Analyses | |
| --- | --- | --- | --- | --- | --- | --- |
|  | *r* | *95% CI* | *r* | *95% CI* | *r* | *95% CI* |
| status-resources | .39 | [.29; .49] | .24 | [-.00; .46] | .47 | [.36; .57] |
| warmth-trustworthiness | .16 | [.04; .27] | .03 | [-.22; .27] | .31 | [.19; .43] |
| vitality-attractiveness | .38 | [.27; .47] | .19 | [-.06; .41] | .44 | [.32; .55] |
| family orientation | .40 | [.29; .49] | .22 | [-.02; .44] | .46 | [.34; .56] |
| intelligence | .22 | [.10; .33] | .12 | -.12; .36] | .37 | [.25; .49] |
| humour | - | - | - | - | .40 | [.27 .51] |
| creativity | .26 | [.14; .37] | .02 | [-.23; .26] | .40 | [.27; .51] |
| adventurousness-confidence | .33 | [.22; .44] | .16 | [-.09; .39] | .47 | [.35; .57] |
| Overall | .32 |  | .14 |  | .42 |  |

## **B.** **Profile Correlation**

Across both samples, we found a normative profile correlation on an item-level of *q̄* = 0.62 (*t* (204) = 19.67, *p* <.001) and a distinctive profile correlation of *q̄* = 0.39 (*t* (204) = 23.82, *p* <.001). For former participants of the Sociosexuality study, we found a normative profile correlation on an item-level of *q̄* = 0.17 (*t* (65) = 2.10, *p* <.020) and a distinctive profile correlation of *q̄* = 0.11 (*t* (65) = 2.34, *p* <.011).

When comparing the profile correlation on an item-level of our main analyses (overall correlation: *r* = .62, *p* <.001; distinctive stability: *r* = .40, *p* < .001) to analyses on former participants of the Sociosexuality Study, we found a smaller overall as well as distinctive stability in participants of the former Sociosexuality study. However, to be noted, our main analyses are based on 58 items, whereas analyses based on former participants of the Sociosexuality Study is based on 12 items only.

## **C.** **Mean-level Changes**

In the sample of former participants of the BSDS, we found increased preferences for the dimensions warmth-trustworthiness, status-resources and decreases for vitality-attractiveness. Paired sample *t*-tests (two-sided) revealed similar results for the dimensions warmth-trustworthiness and status-resources in the analyses of former participants of the Sociosexuality Study (Table S26), whereas the preference for vitality-attractiveness did not show significant changes. Additionally, analyses of former participants of the Sociosexuality Study revealed increased preferences for the dimensions intelligence and family orientation.

**Table S26**

*Means, standard deviations and effect sizes of T1 and T2 importance ratings reported across studies (both samples) and for former participants of the Sociosexuality Study.*

|  | Both samples (*n* = 269) | | | | Sociosexuality Study (*n* =65) | | | |
| --- | --- | --- | --- | --- | --- | --- | --- | --- |
|  | *M*_T1_ (*SD*) | *M*_T2_ (*SD*) | *p* | *d* | *M*_T1_ (*SD*) | *M*_T2_ (*SD*) | *p* | *d* |
| status-resources | 2.86 (.60) | 2.92 (.63) | .10 | .10 | 3.21 (.42) | 3.00 (.69) | =.02 | .29 |
| warmth-trustworthiness | 4.02 (.55) | 4.29 (.56) | < .001 | .37 | 3.58 (.61) | 4.49 (.73) | <.001 | .94 |
| vitality-attractiveness | 3.64 (.54) | 3.59 (.58) | .26 | .07 | 3.64 (.57) | 3.80 (.58) | .08 | .08 |
| family orientation | 3.40 (.93) | 3.56 (1.16) | .03 | .14 | 3.17 (.82) | 3.66 (1.27) | =.004 | .37 |
| intelligence | 3.75 (.62) | 3.94 (.58) | < .001 | .24 | 3.34 (.71) | 4.28 (.70) | <.001 | 1.01 |
| humour | - |  | - | - | - | 3.67 (.54) | - | - |
| creativity | 3.53 (.63) | 3.44 (.65) | .04 | .13 | 3.57 (.79) | 3.35 (.93) | .15 | .18 |
| adventurousness-confidence | 3.25 (.56) | 3.15 (.55) | .01 | .16 | 3.55 (.64) | 3.68 (.85) | .31 | .13 |
| Overall | 3.46 (.31) | 3.52 (.40) | .03 | .13 |  |  |  |  |

*Note.* At T1, importance of humour was only assessed in the BSDS sample but not in the Sociosexuality Study. For the overall T1 and T2 preference, we calculated the mean importance rating of all preference items separately for each timepoint. For the overall correlation we z-transformed each correlation of each preference dimension, calculated the mean z-value and transformed it into a correlation coefficient.

## **D.** **Association of Age**

**Table S27**

*Multilevel models investigating whether age is associated with participants’ preference change for the dimension warmth-trustworthiness, vitality-attractiveness, status-resources and family orientation reported across both initial samples*

|  | warmth-trustworthiness | | | vitality-attractiveness | | | status-resources | | | Family orientation | | |
| --- | --- | --- | --- | --- | --- | --- | --- | --- | --- | --- | --- | --- |
| Predictors | *b* | 95% CI | *p* | *b* | 95% CI | *p* | *b* | 95% CI | *p* | *b* | 95% CI | p |
| (Intercept) | 4.02 | 3.96 – 4.09 | <0.001 | 3.64 | 3.57 – 3.70 | <0.001 | 2.86 | 2.78 – 2.93 | <0.001 | 3.40 | 3.28 – 3.52 | <0.001 |
| time point | 0.27 | 0.18 – 0.35 | <0.001 | -0.04 | -0.12 – 0.03 | 0.249 | 0.07 | -0.01 – 0.15 | 0.095 | 0.16 | 0.03 – 0.30 | 0.015 |
| age at T1 | 0.11 | 0.04 – 0.17 | 0.002 | 0.04 | -0.02 – 0.11 | 0.218 | -0.05 | -0.12 – 0.03 | 0.219 | -0.12 | -0.23 – 0.00 | 0.056 |
| time point * age at T1 | -0.17 | -0.26 – -0.09 | <0.001 | -0.12 | -0.19 – -0.05 | 0.001 | 0.03 | -0.05 – 0.12 | 0.402 | -0.36 | -0.50 – -0.23 | <0.001 |
| Random Effects | | | | | | | | | | | | |
| σ^2^ | 0.25 | | | 0.19 | | | 0.23 | | | 0.61 | | |
| τ_00_ | 0.06 _session_ | | | 0.12 _session_ | | | 0.15 _session_ | | | 0.37 _session_ | | |
| ICC | 0.19 | | | 0.39 | | | 0.39 | | | 0.38 | | |
| N | 269 _session_ | | | 269 _session_ | | | 269 _session_ | | | 269 _session_ | | |
| Observations | 538 | | | 538 | | | 538 | | | 538 | | |
| Marginal R^2^ / Conditional R^2^ | 0.078 / 0.249 | | | 0.014 / 0.399 | | | 0.006 / 0.395 | | | 0.115 / 0.449 | | |

*Note.* *b* = Estimate, 95% CI = 95% confidence interval, *p* = *p*-value. Preferences are predicted with the assessment time point (0 = T1, 1 = T2), age at T1 (*z*-standardised) and their interaction. A random effect is specified for each person.

**Table S28**

*Multilevel models investigating whether age is associated with participants’ preference change for the dimension intelligence, creativity, humour and adventurousness-confidence reported across both initial samples*

|  | intelligence | | | creativity | | | humour | | | adventurousness-confidence | | |
| --- | --- | --- | --- | --- | --- | --- | --- | --- | --- | --- | --- | --- |
| Predictors | *b* | 95% CI | *p* | *b* | 95% CI | *p* | *b* | 95% CI | *p* | *b* | 95% CI | *p* |
| (Intercept) | 3.75 | 3.68 – 3.82 | <0.001 | 3.53 | 3.45 – 3.61 | <0.001 | 3.44 | 3.36 – 3.52 | <0.001 | 3.25 | 3.19 – 3.32 | <0.001 |
| time point | 0.18 | 0.10 – 0.27 | <0.001 | -0.10 | -0.19 – -0.00 | 0.040 | 0.06 | -0.02 – 0.15 | 0.153 | -0.10 | -0.18 – -0.02 | 0.011 |
| age at T1 | 0.05 | -0.02 – 0.12 | 0.187 | 0.01 | -0.06 – 0.09 | 0.751 | -0.09 | -0.17 – -0.02 | 0.019 | -0.12 | -0.18 – -0.05 | <0.001 |
| time point * age at T1 | -0.16 | -0.25 – -0.07 | <0.001 | -0.01 | -0.10 – 0.08 | 0.837 | -0.06 | -0.14 – 0.03 | 0.188 | 0.04 | -0.04 – 0.12 | 0.307 |
| Random Effects | | | | | | | | | | | | |
| σ^2^ | 0.27 | | | 0.31 | | | 0.20 | | | 0.21 | | |
| τ_00_ | 0.08 _session_ | | | 0.11 _session_ | | | 0.11 _session_ | | | 0.09 _session_ | | |
| ICC | 0.23 | | | 0.26 | | | 0.37 | | | 0.31 | | |
| N | 269 _session_ | | | 269 _session_ | | | 269 _session_ | | | 269 _session_ | | |
| Observations | 538 | | | 538 | | | 473 | | | 538 | | |
| Marginal R^2^ / Conditional R^2^ | 0.044 / 0.268 | | | 0.006 / 0.262 | | | 0.056 / 0.404 | | | 0.039 / 0.341 | | |

*Note.* *b* = Estimate, 95% CI = 95% confidence interval, *p* = *p*-value. Preferences are predicted with the assessment time point (0 = T1, 1 = T2), age at T1 (*z*-standardised) and their interaction. A random effect is specified for each person.

**Table S29**

*Multilevel models investigating whether age is associated with participants’ preference change for the dimension warmth-trustworthiness, vitality-attractiveness, status-resources and family orientation reported for former participants of the Sociosexuality Study.*

|  | warmth-trustworthiness | | | vitality-attractiveness | | | status-resources | | | Family orientation | | |
| --- | --- | --- | --- | --- | --- | --- | --- | --- | --- | --- | --- | --- |
| Predictors | *b* | 95% CI | *p* | *b* | 95% CI | *p* | *b* | 95% CI | *p* | *b* | 95% CI | *p* |
| (Intercept) | 3.58 | 3.42 – 3.75 | <0.001 | 3.64 | 3.50 – 3.78 | <0.001 | 3.21 | 3.07 – 3.35 | <0.001 | 3.17 | 2.91 – 3.43 | <0.001 |
| time point | 0.91 | 0.68 – 1.14 | <0.001 | 0.16 | -0.02 – 0.34 | 0.074 | -0.21 | -0.38 – -0.03 | 0.020 | 0.49 | 0.17 – 0.81 | 0.003 |
| age at T1 | -0.03 | -0.19 – 0.14 | 0.757 | -0.01 | -0.15 – 0.13 | 0.936 | 0.01 | -0.13 – 0.15 | 0.889 | 0.17 | -0.09 – 0.43 | 0.212 |
| time point * age at T1 | -0.12 | -0.35 – 0.11 | 0.295 | -0.13 | -0.31 – 0.05 | 0.147 | 0.83 | -0.26 – 0.09 | 0.358 | -0.30 | -0.63 – 0.02 | 0.065 |
| Random Effects | | | | | | | | | | | | |
| σ^2^ | 0.44 | | | 0.27 | | | 0.26 | | | 0.88 | | |
| τ_00_ | 0.01 _session_ | | | 0.06 _session_ | | | 0.07 _session_ | | | 0.26 _session_ | | |
| ICC | 0.02 | | | 0.19 | | | 0.22 | | | 0.23 | | |
| N | 65 _session_ | | | 65 _session_ | | | 65 _session_ | | | 65 _session_ | | |
| Observations | 130 | | | 130 | | | 130 | | | 130 | | |
| Marginal R^2^ / Conditional R^2^ | 0.328 / 0.341 | | | 0.046 / 0.230 | | | 0.039 / 0.247 | | | 0.069 / 0.281 | | |

*Note.* *b* = Estimate, 95% CI = 95% confidence interval, *p* = *p*-value. Preferences are predicted with the assessment time point (0 = T1, 1 = T2), age at T1 (*z*-standardised) and their interaction. A random effect is specified for each person.

**Table S30**

*Multilevel models investigating whether age is associated with participants’ preference change for the dimension intelligence, creativity and adventurousness-confidence reported for former participants of the Sociosexuality Study*

|  | intelligence | | | creativity | | | adventurousness-confidence | | |
| --- | --- | --- | --- | --- | --- | --- | --- | --- | --- |
| Predictors | *b* | 95% CI | *p* | *b* | 95% CI | *p* | *b* | 95% CI | *p* |
| (Intercept) | 3.34 | 3.17 – 3.51 | <0.001 | 3.57 | 3.36 – 3.78 | <0.001 | 3.55 | 3.38 – 3.73 | <0.001 |
| time point | 0.94 | 0.71 – 1.17 | <0.001 | -0.22 | -0.51 – 0.08 | 0.153 | 0.12 | -0.12 – 0.36 | 0.313 |
| age at T1 | -0.05 | -0.22 – 0.12 | 0.549 | 0.04 | -0.17 – 0.25 | 0.702 | -0.18 | -0.36 – -0.01 | 0.044 |
| time point * age at T1 | -0.05 | -0.28 – 0.18 | 0.672 | -0.00 | -0.30 – 0.29 | 0.976 | -0.00 | -0.24 – 0.24 | 0.989 |
| Random Effects | | | | | | | | | |
| σ^2^ | 0.44 | | | 0.74 | | | 0.48 | | |
| τ_00_ | 0.06 _session_ | | | 0.01 _session_ | | | 0.05 _session_ | | |
| ICC | 0.11 | | | 0.02 | | | 0.10 | | |
| N | 65 _session_ | | | 65 _session_ | | | 65 _session_ | | |
| Observations | 130 | | | 130 | | | 130 | | |
| Marginal R^2^ / Conditional R^2^ | 0.315 / 0.392 | | | 0.017 / 0.034 | | | 0.066 / 0.160 | | |

Note. b = Estimate, 95% CI = 95% confidence interval, p = p-value. Preferences are predicted with the assessment time point (0 = T1, 1 = T2), age at T1 (*z*-standardised) and their interaction. A random effect is specified for each person.

## **E.** **Association of Parenthood**

**Rank-Order stability for participants with and without children**

We decided not to perform these analyses for former participants of the Sociosexuality Study because of the small sample size. We would have needed to divide the already small sample of 65 participants, in an even much smaller subgroup of participants with vs. without children. These analyses would not provide any reliable insight. Therefore we only report rank-order stabilities for participants with vs. without children across both studies in Table S31.

**Table S31**

*Means, standard deviations, effect sizes and correlations of T1 and T2 importance ratings separately for participants with and without children*

|  | participants with children at T2 (*n* = 155) | | | | | participants without children at T2 (*n* = 88) | | | | |
| --- | --- | --- | --- | --- | --- | --- | --- | --- | --- | --- |
| dimension | *M_T1 with_*  (*SD _with_*) | *M_T2 with_* (*SD_with_*) | *p_with_* | *d_with_* | *r_with_* | *M_T1 without_* (*SD_without_*) | *M_T2 without_* (*SD_without_*) | *p_without_* | *d_without_* | *r_without_* |
| status-resources | 2.83 (.63) | 2.93 (.59) | .16 | .14 | .32 | 2.87 (.59) | 2.87 (.70) | .98 | .00 | .46 |
| warmth-trustworthiness | 4.00 (.57) | 4.37 (.54) | <.001 | .48 | .09 | 3.97 (.57) | 4.20 (.50) | =.007 | .39 | .39 |
| vitality-attractiveness | 3.65 (.55) | 3.62 (.58) | .67 | .04 | .40 | 3.61 (.54) | 3.61 (.53) | .97 | .01 | .44 |
| family orientation | 3.68 (.74) | 4.22 (.72) | <.001 | .56 | .12 | 2.86 (1.05) | 2.54 (1.10) | .06 | .25 | .24 |
| intelligence | 3.75 (.64) | 3.99 (.54) | <.001 | .30 | .10 | 3.76 (.64) | 3.92 (.60) | .10 | .25 | .43 |
| humour | 3.53 (.48) | 3.53 (.59) | .97 | .00 | .28 | 3.28 (.53) | 3.41 (.62) | .22 | .22 | .52 |
| creativity | 3.53 (.64) | 3.43 (.64) | .16 | .13 | .20 | 3.51 (.65) | 3.46 (.61) | .63 | .07 | .37 |
| adventurousness-confidence | 3.28 (.57) | 3.20 (.54) | .18 | .13 | .30 | 3.23 (.61) | 3.09 (.60) | .15 | .20 | .36 |

*Note.* Results for the dimension humour are only based on participants and items of the former BSDS.

## **F.** **Insight into Preference Change**

In the analyses of former participants of the Sociosexuality Study, we found a mean accuracy of *r* = .29, which is comparable to the results of our main analyses (mean accuracy in BSDS .20). Similar to our main analyses, across dimensions, coefficients ranged considerably from *r* = .11 to *r* = .50 (Table S32).

**Table S32**

Participant’s actual and perceived change of each preference dimension as well as the correlation across participants of both former studies.

|  | actual change | | perceived change | | responses of perceived change (%) | | | | | insight | | |
| --- | --- | --- | --- | --- | --- | --- | --- | --- | --- | --- | --- | --- |
|  | *M* | *SD* | *M* | *SD* | -2 | -1 | 0 | 1 | 2 | *r* | *p* | 95% CI |
| status-resources | 0.07 | 0.68 | -0.06 | 0.80 | 4.0 | 20.4 | 55.0 | 18.2 | 2.2 | 0.15 | =.01 | [0.03;0.27] |
| warmth-trustworthiness | 0.27 | 0.72 | 0.48 | 0.73 | 0.0 | 3.3 | 56.5 | 29.4 | 10.8 | 0.20 | <.001 | [0.08;0.31] |
| vitality-attractiveness | -0.04 | 0.62 | -0.26 | 0.71 | 2.6 | 32.0 | 55.8 | 8.2 | 1.5 | 0.23 | <.001 | [0.11;0.34] |
| family orientation | 0.16 | 1.17 | 0.24 | 120 | 11.2 | 11.9 | 36.1 | 23.8 | 17.1 | 0.50 | <.001 | [0.40;0.58] |
| intelligence | 0.18 | 0.75 | 0.24 | 0.66 | 1.1 | 3.7 | 70.3 | 19.7 | 5.2 | 0.14 | =.02 | [0.02;0.26] |
| humour | 0.04 | 0.63 | 0.10 | 0.64 | 0.7 | 10.8 | 68.4 | 17.5 | 2.6 | 0.14 | =.04 | [0.01;0.28] |
| creativity | -0.10 | 0.78 | -0.11 | 0.76 | 3.0 | 24.5 | 54.3 | 16.7 | 1.5 | 0.25 | <.001 | [0.13;0.36] |
| adventurousness-confidence | -0.10 | 0.64 | -0.16 | 0.80 | 1.5 | 33.1 | 48.0 | 14.5 | 3.0 | 0.14 | =.02 | [0.02;0.26] |

**Table S33**

Participant’s actual and perceived change of each preference dimension as well as the correlation in the sample of former participants of the Sociosexuality Study.

|  | actual change | | perceived change | | responses of perceived change (%) | | | | | insight | | |
| --- | --- | --- | --- | --- | --- | --- | --- | --- | --- | --- | --- | --- |
|  | *M* | *SD* | *M* | *SD* | -2 | -1 | 0 | 1 | 2 | *r* | *p* | 95% CI |
| status-resources | -.21 | .72 | .09 | .72 | 1.54 | 13.85 | 61.54 | 20.00 | 3.08 | .39 | =.001 | [.16; .58] |
| warmth-trustworthiness | .91 | .94 | .62 | .76 | 0.00 | 3.08 | 46.15 | 36.92 | 13.85 | .11 | =.370 | [-.13; .35] |
| vitality-attractiveness | .16 | .73 | -.22 | .67 | 1.54 | 29.23 | 60.00 | 7.69 | 1.54 | .34 | =.006 | [.10; .54] |
| family orientation | .49 | 1.35 | .72 | 1.07 | 6.15 | 1.54 | 32.31 | 33.85 | 26.15 | .50 | <.001 | [.29; .66] |
| intelligence | .94 | .93 | .34 | .76 | 1.54 | 4.62 | 60.00 | 26.15 | 7.69 | .12 | =.347 | [-.13; .35] |
| humour | - | - | .28 | .65 | 6.15 | 64.62 | 24.62 | 4.62 | 6.15 | - | - | - |
| creativity | -.22 | 1.21 | -.08 | .82 | 4.62 | 21.54 | 52.31 | 20.00 | 1.54 | .25 | =.042 | [.01; .47] |
| adventurousness-confidence | .12 | .98 | -.14 | .79 | 1.54 | 32.31 | 46.15 | 18.46 | 1.54 | .25 | =.048 | [.00; .46] |

#

#

# **S4. Deviations from our Preregistration**

**Table S34**

*Description and explanation of all deviations from our preregistration*

| Pre-registration | Manuscript | Explanation |
| --- | --- | --- |
| We preregistered that our final sample would consist out of two initial samples (BSDS and Sociosexuality Study). | We analysed both samples separately, whereas we only report analyses based on the former BSDS in our main manuscript. Analyses based on the former sample of the Sociosexuality Study are reported in our supplement.  The consequence of this change is a different number of items which contribute to the mean each preference dimension. | Assessment of T1 ideal partner preferences differed tremendously between the two initial samples. Because in the Sociosexuality Study T1 preferences were assessed insufficiently, we realised that combining both samples would be inappropriate.  We decided to only report results of the sample of former participants of the Sociosexuality Study as part of the supplemental analyses because the sample is too small and does not provide adequate power to draw reliable conclusions. |
| When investigating the relationship of age and mean-level changes, we specified multilevel models, separately for each preference dimension. Initially, we pre-registered that we would use a 2 (age group) x 2 (time point) repeated measures ANOVA separately for the dimension status-resources and family-orientation. Additionally, we pre-registered to explore all other dimensions using a 8 (preference dimension) x 2 (age group) x 2 (time point) MANOVA. | For each preference dimension, we ran a multilevel model in which we predicted participants’ preferences with the time point (0 = T1, 1 = T2), age (*z*-standardised) and their interaction, while including a random effect for participants because of the repeated measurement. | Running multilevel models allowed us to analyse the variable age continuously, which we deem to be more appropriate. |
| When investigating whether the preference for status-resources changes with the immediacy or existence of having children, we preregistered to compare mean levels using a 2 (group) x 2 (time point) repeated measures ANOVA next to the comparison of differences in correlation coefficients between the groups. We preregistered to additionally compare mean levels. | We compared correlation coefficients as pre-registered. However, instead of a repeated measures ANOVA, we specified multilevel models for the exploratory analyses in changes in mean-levels.  We predicted ideals with the time point (0 = T1, 1 = T2), whether participants have children (0 = without children, 1 = with children), their interaction, and a random effect taking the repeated measurement into account. | A multilevel model is a more appropriate approach of analysing the data compared to a repeated-measures ANOVA. |

# **S6. Conference Presentation**

Portions of this research have been presented at the 21st Annual Meeting of the Society for Personality and Social Psychology in New Orleans (February 2020) and online at the International Association for Relationship Research (July, 2021).

# **S7. Author Contributions**

JCD, LP and TMG planned T2 data collection. LP planned and collected T1 data. JDC collected T2 data, analysed T1 and T2 data and wrote initial draft of the manuscript. LP and TMG supervised the project. TMG, JS and LP provided feedback and critically revised the manuscript. All authors read and approved the final manuscript

# **S8. References**

Borkenau, P., & Ostendorf, F. (1993). NEO-Fünf-Faktoren Inventar nach Costa und McCrae [NEO Five Factor Inventory after Costa and McCrae]. Göttingen, Germany: Hogrefe.

Fletcher, G. J. O., Simpson, J. A., & Thomas, G. (2000). Ideals, perceptions, and evaluations in early relationship development. *Journal of Personality and Social Psychology*, *79*(6), 933–940. <https://doi.org/10.1037/0022-3514.79.6.933>

Gerlach, T. M., Arslan, R. C., Schultze, T., Reinhard, S. K., & Penke, L. (2019). Predictive validity and adjustment of ideal partner preferences across the transition into romantic relationships. *Journal of Personality and Social Psychology*, *116*(2), 313–330. https://doi.org/10.1037/pspp0000170

Lang, F. R., Lüdtke, O., & Asendorpf, J. B. (2001). Testgüte und psychometrische Äquivalenz der deutschen Version des Big Five Inventory (BFI) bei jungen, mittelalten und alten Erwachsenen. *Diagnostica*, *47*(3), 111-121.<http://dx.doi.org/10.1026/0012-1924.47.3.111>

Mackinnon, S., Curtis, R., & O'Connor, R. (2022). A Tutorial in longitudinal measurement invariance and cross-lagged panel models Using lavaan. *Meta-Psychology*, *6*, MP.2020.2595. <https://doi.org/10.15626/MP.2020.2595>

Penke, L., & Asendorpf, J. B. (2008). Beyond global sociosexual orientations: A more differentiated look at sociosexuality and its effects on courtship and romantic relationships. *Journal of Personality and Social Psychology*, *95*(5), 1113–1135.<https://doi.org/10.1037/0022-3514.95.5.1113>
